# Rational Extension of the Ribosome Biogenesis Pathway Using Network-Guided Genetics

**DOI:** 10.1371/journal.pbio.1000213

**Published:** 2009-10-06

**Authors:** Zhihua Li, Insuk Lee, Emily Moradi, Nai-Jung Hung, Arlen W. Johnson, Edward M. Marcotte

**Affiliations:** 1 Center for Systems and Synthetic Biology, Institute for Cellular and Molecular Biology, University of Texas, Austin, Texas, United States of America; 2 Department of Biotechnology, College of Life science and Biotechnology, Yonsei University, 134 Shinchon-dong, Seodaemun-ku, Seoul 120-749, South Korea; 3 Section of Molecular Genetics and Microbiology, Institute for Cellular and Molecular Biology, University of Texas, Austin, Texas, United States of America; 4 Department of Chemistry and Biochemistry, University of Texas, Austin, Texas, United States of America; University of California, Berkeley, United States of America

## Abstract

Biogenesis of ribosomes is an essential cellular process conserved across all eukaryotes and is known to require >170 genes for the assembly, modification, and trafficking of ribosome components through multiple cellular compartments. Despite intensive study, this pathway likely involves many additional genes. Here, we employ network-guided genetics—an approach for associating candidate genes with biological processes that capitalizes on recent advances in functional genomic and proteomic studies—to computationally identify additional ribosomal biogenesis genes. We experimentally evaluated >100 candidate yeast genes in a battery of assays, confirming involvement of at least 15 new genes, including previously uncharacterized genes (*YDL063C*, *YIL091C*, *YOR287C*, *YOR006C/TSR3*, *YOL022C/TSR4*). We associate the new genes with specific aspects of ribosomal subunit maturation, ribosomal particle association, and ribosomal subunit nuclear export, and we identify genes specifically required for the processing of 5S, 7S, 20S, 27S, and 35S rRNAs. These results reveal new connections between ribosome biogenesis and mRNA splicing and add >10% new genes—most with human orthologs—to the biogenesis pathway, significantly extending our understanding of a universally conserved eukaryotic process.

## Abstract 2

Gene networks are an efficient route for associating candidate genes with biological processes. Here, networks are used to discover more than 15 new genes for ribosomal subunit maturation, rRNA processing, and ribosomal export from the nucleus.

## Author Summary

Ribosomes are the extremely complex cellular machines responsible for constructing new proteins. In eukaryotic cells, such as yeast, each ribosome contains more than 80 protein or RNA components. These complex machines must themselves be assembled by an even more complex machinery spanning multiple cellular compartments and involving perhaps 200 components in an ordered series of processing events, resulting in delivery of the two halves of the mature ribosome, the 40S and 60S components, to the cytoplasm. The ribosome biogenesis machinery has been only partially characterized, and many lines of evidence suggest that there are additional components that are still unknown. We employed an emerging computational technique called network-guided genetics to identify new candidate genes for this pathway. We then tested the candidates in a battery of experimental assays to determine what roles the genes might play in the biogenesis of ribosomes. This approach proved an efficient route to the discovery of new genes involved in ribosome biogenesis, significantly extending our understanding of a universally conserved eukaryotic process.

## Introduction

In eukaryotic cells, the synthesis of ribosomes is a complex process involving several hundred genes whose functions span transcription of precursor ribosomal ribonucleic acids (pre-rRNAs), processing of pre-rRNAs, assembly of ribosomal proteins (r-proteins) with pre-rRNAs, and nuclear export of the ribosomal particles [1]–[6]. Ribosome biogenesis is an essential process, with mutations of ribosome biogenesis genes either causing lethality or increasing susceptibility to cancer—e.g., bone marrow failure and leukemia [7] or breast cancer [8]. This pathway has been extensively studied over the past 30–40 y, and a broad picture of the major events is known for the yeast *Saccharomyces cerevisiae*. First, 35S polycistronic pre-rRNA is transcribed from the ribosomal deoxyribonucleic acid (rDNA) repeat by RNA polymerase I in the nucleolus. During transcription, the small-subunit processome and some small-subunit r-proteins assemble onto the 35S pre-rRNA to form a 90S particle. The 35S pre-rRNA is cleaved to release the pre-40S particle, which contains a 20S pre-rRNA. The pre-60S complex assembles on the rest of the transcript, and both subunits are further processed in the nucleus and independently exported through the nuclear pore complex (NPC) to the cytoplasm, where they undergo further maturation—e.g., cleavage of 20S pre-rRNA to 18S rRNA. The mature small subunit contains 32 proteins and 18S rRNA, while the large subunit contains 46 proteins and three rRNAs: 5.8S, 25S, both derived from the 35S precursor, and 5S, which is transcribed separately by RNA polymerase III.

Ribosome biogenesis is a temporally and spatially dynamic process requiring coordination of many trans-acting factors at different stages along the pathway, including at least 170 protein factors that act to modify and cleave pre-rRNAs and help to assemble and export ribosomal particles [5],[9]. Many of these protein factors were first identified by yeast genetics. Later, biochemical purifications coupled with mass spectrometric analysis greatly expanded the number of known factors [10]–[16]. In addition, a large-scale effort using oligonucleotide microarrays identified 115 mutants that exhibited pre-rRNA processing defects, and 10 new genes were confirmed to affect pre-rRNA processing [17]. Despite these intensive studies, new ribosome biogenesis genes are still emerging, and recent computational analysis suggests that over 200 genes constitute the ribosome biogenesis regulon [18], indicating that the genes in this fundamental cellular pathway have not been completely identified.

We asked if recent functional genomic and proteomic studies could be applied in a predictive fashion to identify additional ribosomal biogenesis genes. In particular, functional networks of genes have been reconstructed, incorporating literally millions of experimental observations into probabilistic networks indicating genes likely to work together in cells. The emerging technique of network-guided genetics (e.g., [19],[20]) leverages such networks to computationally associate candidate genes with a biological process of interest, much as a genetic screen might do. We used such a probabilistic gene network [21] to predict the genes most likely to participate in yeast ribosome biogenesis based on connectivity to known ribosomal biogenesis genes, and we present here experimental confirmation of at least 15 new genes affecting ribosome biogenesis. Beyond providing new insights into ribosome biogenesis, this study therefore also represents one of the most extensive experimental studies to date of the principle of network-guided genetics, which we demonstrate to be a powerful approach for rational discovery of candidate genes, applicable to diverse biological processes.

## Results

### Using Network-Guided Genetics to Predict New Ribosome Biogenesis Genes

**Figure 1:**
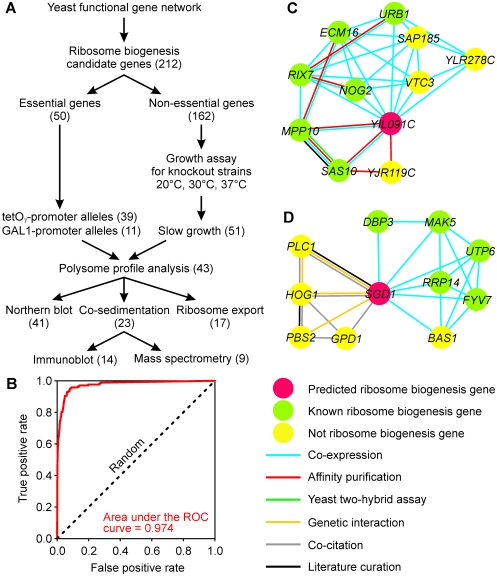
Overview of the analysis. (A) A yeast functional-gene network reconstructed from diverse functional genomic and proteomic data [21] was employed to predict genes for ribosome biogenesis. For nonessential genes, growth assays of the deletion mutants under different temperature conditions (20°C, 30°C, and 37°C) were used to identify conditional growth defects, and polysome profiles of these strains were collected under slow-growth conditions. For essential genes, mutants with conditional alleles were subjected to polysome profile analyses after depleting the encoded proteins. Genes affecting the ratio of free 40S to free 60S ribosomal subunits upon deletion of the gene or depletion of the encoded protein were further analyzed by co-sedimentation analyses to assign possible protein association with pre-ribosomal particles, by using Northern blots to assay pre-rRNA processing defects, and by ribosomal subunit export assays. Numbers in parentheses are counts of genes implicated in ribosome biogenesis by each analysis. (B) Assessment of the network-based predictability of ribosome biogenesis genes. The ROC curve (red line) shows cross-validated recovery of known ribosome biogenesis genes based on their network connectivity to one another. True positive ribosome biogenesis genes were manually curated based on Gene Ontology annotation. The network-based prediction is considerably stronger than random expectation (dashed line). (C) and (D) show the top 10 network connections for two predicted ribosome biogenesis genes, *YIL091C* and *SGD1*.

In general, we expect genes of ribosome biogenesis to be coordinately expressed, to physically or genetically interact with each other, to show common subcellular localization, and so on. Many such associations have been observed in high-throughput experiments in yeast, but these data suffer from false-positive and false-negative observations. Nonetheless, the appropriate analyses of such data should rationally prioritize candidate ribosome biogenesis genes. We therefore constructed a computational predictor of ribosome biogenesis genes based on analysis of functional genomics, proteomics, and comparative genomics datasets that had been combined into a probabilistic gene network [21] covering about 95% of yeast proteome (Figure 1A). This network employs a probabilistic scoring scheme to quantitatively integrate heterogeneous functional genomic and proteomic datasets, including mRNA-expression data across different conditions, protein-protein interaction datasets derived from literature curation, high-throughput yeast two-hybrid assay, affinity purification coupled with mass spectrometry, genetic interaction data, and *in silico* interaction datasets [21]. We calculated the *naïve* Bayesian probability that each yeast gene will belong to the ribosome biogenesis pathway based on gene connectivity information in the gene network—i.e., “guilt-by-association” [22],[23] with known ribosome biogenesis genes. Ribosome biogenesis genes were highly connected and predictable in this gene network, as shown by a plot of cross-validated true-positive versus false-positive prediction rates (ROC plot; Figure 1B). From the top-scoring genes, 212 candidates were manually selected based on expert knowledge for experimental validation (Table S1).

### Conditional Growth Phenotypic Analysis for Nonessential Genes

The synthesis of ribosomes is essential for cell growth and survival, and most genes involved in ribosome biogenesis are either essential or required for normal growth rates. In our list of candidate ribosome biogenesis genes, 50 genes are essential, and 162 genes are nonessential under standard laboratory culture conditions [24]. We thus performed growth assays for each strain with a deletion of one of the 162 nonessential genes under three temperature conditions: 20°C, 30°C, and 37°C (Figure S1). Of these, 51 mutants with constitutive or conditional slow-growth phenotypes were identified. These mutants and 50 mutants carrying conditional essential alleles were investigated further (Figure 1A).

### Verifying Ribosomal Subunit Biogenesis Defects by Polysome Profile Analysis

For each of the selected 101 mutants, we tested for gross ribosome biogenesis defects by measuring the proportions of free 40S, 60S, and 80S subunits, as well as polysomes, in the mutant strains. After cleavage of the pre-40S particle from the 35S transcript, the syntheses of 40S and 60S subunits are largely independent [6]. Depletion of the factors required for the synthesis of one subunit usually does not significantly affect synthesis of the other subunit [25], resulting in a change in the ratio of 40S to 60S, which is most evident in the free subunit pools in the cell. In addition, a reduction in the amount of 60S subunits can lead to a translation initiation defect, with 40S subunits awaiting 60S subunits to form 80S ribosomes. These stalled 40S subunits are observable as halfmer polysomes in a polysome profile [26]. Polysome profiles are generated by separating the ribosomal subunits and different-sized polysomes through a continuous sucrose density gradient and monitoring the absorbance of nucleic acids along the sucrose gradient [27]. We analyzed polysome profiles for the 50 mutants carrying conditional alleles controlled by either a tetracycline-regulatable (tetO_7_) promoter [28] or a GAL1 promoter and for the 51 nonessential gene deletion mutants with conditional growth defects.

**Figure 2:**
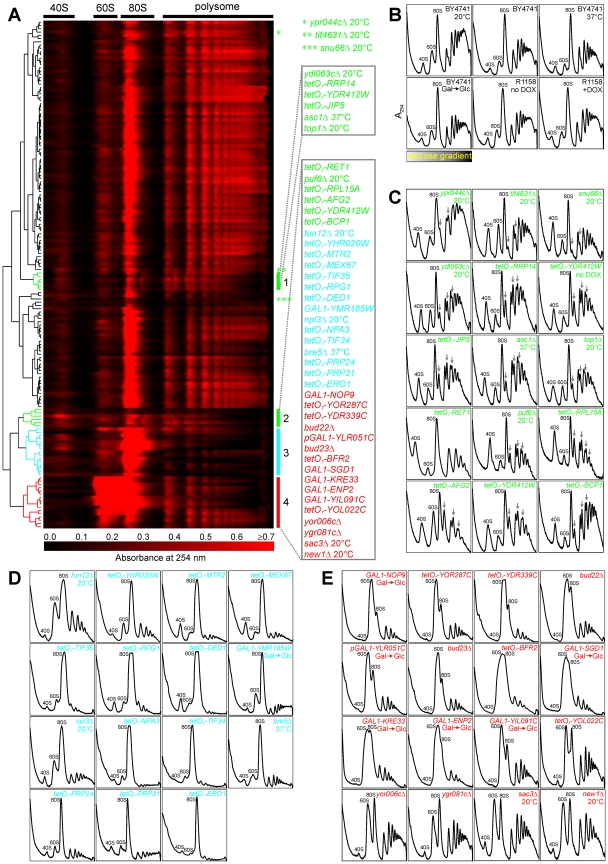
Ribosome biogenesis defects were confirmed and classified by polysome profiles of mutant strains. (A) Hierarchical clustering of mutant polysome profiles (rows), with clusters 1 and 2 representing mutants with 60S subunit biogenesis defects (green), cluster 3 displaying translation defects (cyan), and cluster 4 displaying 40S subunit biogenesis defects (red). Three additional mutants with 60S subunit biogenesis defects are labeled with stars. Each row corresponds to the polysome profile of a single strain, plotting nucleic acid absorbance as a function of position in a sucrose density gradient. Strains were cultured at 30°C unless otherwise indicated. Mutants with tetO_7_-promoter alleles were cultured in medium with 10 µg/ml doxycycline (+DOX) unless indicated with no DOX. Mutants with GAL1-promoter alleles were first cultured in medium with galactose (Gal) as the carbon source and then diluted into medium with dextrose (Glc) as the carbon source. (B) Polysome profiles of wild-type strains cultured under assayed conditions. BY4741 is the control strain for the nonessential gene-deletion mutants and mutants with GAL1-promoter alleles. R1158 is the control strain for the mutants with tetO_7_-promoter alleles. Peaks corresponding to 40S and 60S ribosomal subunits and 80S monosomes in the polysome profiles are labeled. (C, D, and E) Polysome profiles of mutants with 60S subunit biogenesis defects, translation defects, and 40S subunit biogenesis defects. Gray arrows indicate halfmer polysome peaks.

Including controls, over 150 polysome profiles were generated. In order to compare different profiles and perform multivariate analyses such as clustering, we computationally aligned each profile to a reference wild-type profile by using a correlation-optimized warping (COW) algorithm [29], which corrects for peak shifts of ribosome subunits and polysomes due to minor variations in sucrose density gradients. Similar polysome profiles were grouped together using hierarchical clustering [30]. From the clustergram, the signals corresponding to the ribosomal subunits, monosomes, polysomes, and halfmer polysomes were clearly identifiable (Figure 2A). Importantly, nearly half of the tested mutants showed clear ribosome biogenesis defects by this analysis. This is a much higher ratio than the ∼1/30 expected by chance, indicating the strong enrichment for true ribosome biogenesis genes provided by the network-guided genetics.

Several sets of mutants exhibited grossly similar biogenesis defects, detectable as coherent groups in the clustergram. Most of the profiles with high 40S to 60S ratios and halfmer peaks were in clusters 1 and 2, which represent 60S biogenesis defects (Figure 2C). Cluster 3 represents profiles from mutants showing protein translation defects (Figure 2D), some of which also affected the ratio of 40S to 60S ribosomal subunits when compared to wild-type strains (Figure 2B). It is noteworthy that the translation-initiation factor mutants, including *fun12Δ*, *tetO_7_-TIF35*, *tetO_7_-TIF34*, *tetO_7_-RPG1*, and *tetO_7_-DED1*, did not display the same defects, indicating that the observed ribosome biogenesis defects are not simply a general effect of inhibition of translation. The profiles with low 40S to 60S ratios were in cluster 4, which suggests 40S biogenesis defects (Figure 2E). The polysome profiles from three mutants (*ypr044cΔ*, *tif4631Δ*, and *snu66Δ*) were not clustered with 60S biogenesis clusters 1 and 2, although they showed halfmer polysomes (Figure 2A, 2C). Some mutants showed only subtle defects, and their profiles were interspersed among wild-type-like profiles during clustering (Figure S2). The polysome profiles provided initial suggestions about the function of these genes in ribosome biogenesis and translation. We further investigated 43 mutants that exhibited altered 40S to 60S ratios compared to wild-type strains (Table S1 and Figure 1A).

### Mapping Physical Association by Co-Sedimentation Analysis on Sucrose Density Gradients

Most ribosome biogenesis factors associate with pre-ribosomal particles [3]. In order to distinguish factors associated with pre-40S particles from factors associated with pre-60S particles, we applied both a classical immunoblot approach and a novel mass-spectrometry-based approach in order to assess sedimentation patterns of potential ribosome biogenesis factors in sucrose density gradients (Figure 1A).

#### Sedimentation patterns of ribosome biogenesis factors by immunoblots

**Figure 3:**
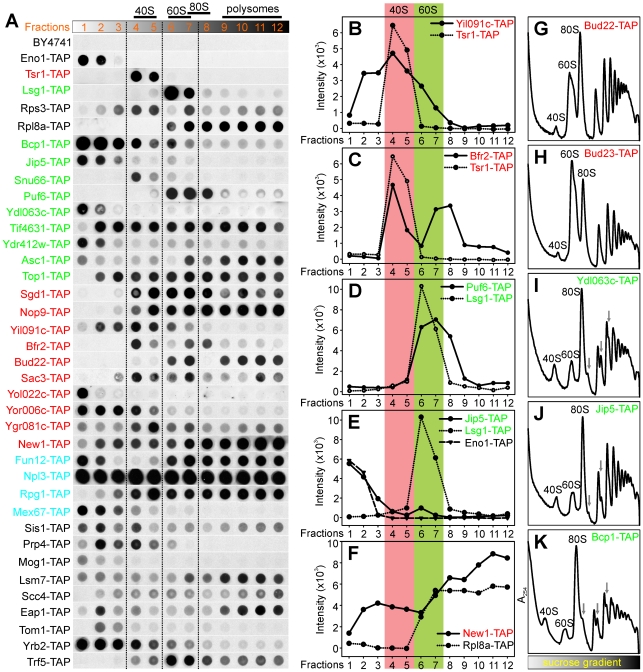
Physical association of candidate proteins with ribosomal subunits was measured by co-sedimentation and immunoblot. (A) Immunoblots of fractions collected from sucrose density gradients for strains carrying TAP-tagged gene alleles. Fractions 4 and 5, 6 and 7, 7 and 8, and 9–12 correspond to the 40S, 60S, 80S, and polysome peaks in the sucrose density gradients, respectively. BY4741 is the negative control, and Tsr1-TAP and Lsg1-TAP are the positive controls for 40S subunit biogenesis factors and 60S subunit biogenesis factors, respectively. Rps3-TAP and Rpl8a-TAP show the locations of small and large ribosomal subunits in the sucrose density gradient, respectively, whereas Eno1-TAP represents the proteins that do not co-sediment with ribosomes. (B–F) show quantitation of the immunoblots for Yil091c-TAP, Bfr2-TAP, Puf6-TAP, Jip5-TAP, and New1-TAP. (G–K) show polysome profile defects for several TAP-tagged strains.

We first asked if epitope-tagged versions of the candidate biogenesis proteins co-sedimented with pre-ribosome particles, which would support physical association with the particles. Strains carrying tandem-affinity purification (TAP)-tagged alleles for 32 of the 43 ribosome biogenesis candidates with polysome profile defects were available [31] and were used to prepare samples for sucrose density gradients. Fractions of each sucrose gradient were collected and analyzed for the TAP-tagged protein by immunoblot (Figure 3A), and the relative abundance of each tagged protein within each fraction was quantified with several examples shown in Figure 3B–3F [32]. We expected 40S biogenesis factors would mainly distribute in the free 40S fractions (e.g., Tsr1-TAP in Figure 3A) and/or 90S fractions, whereas 60S biogenesis factors would mainly distribute in the free 60S fractions (e.g., Lsg1-TAP in Figure 3A). The r-proteins would be expected to be found in the 40S or 60S fractions as well as the monosome and polysome fractions (e.g., Rps3-TAP and Rpl8a-TAP in Figure 3A). In contrast, Eno1p, a cytosolic metabolic enzyme not expected to interact with ribosomes, distributed in the low-density fractions and did not overlap in sedimentation with ribosomes (Figure 3A). We did not detect background signals from the wild-type un-tagged control strain under these experimental conditions (BY4741 in Figure 3A).

As expected, many of the candidate ribosome biogenesis factors sedimented in either 40S or 60S fractions. Yil091cp, an uncharacterized protein [33], was enriched in 40S fractions (Figure 3B), consistent with a role in 40S biogenesis based on polysome profile analysis (Figure 2E). Bfr2p was enriched in 40S fractions and 90S fractions (overlapping with 80S), which suggests that this protein exists in both 40S and 90S pre-ribosomes (Figure 3C). Puf6p sedimented in 60S fractions (Figure 3D), supporting the 60S biogenesis defects observed in the polysome profile of *puf6Δ* (Figure 2C), and consistent with a previous network-based identification of Puf6p as a 60S biogenesis factor [21]. Nop9p, a nucleolus-localized protein [34], was enriched not only in 40S fractions but also across all high-density fractions (Figure 3A), and similar sedimentation patterns were observed for Sgd1p and Top1p (Figure 3A). Deletion of *BUD22* caused a 40S subunit synthesis defect (Figure 2E), but the Bud22 protein co-sedimented with 60S/80S and high-density fractions (Figure 3A). This discrepancy is not unique for *BUD22*: The ribosome biogenesis factor Has1p co-sediments with 60S but mostly affects 40S subunit synthesis upon depletion of the protein [35]. Bud22p thus likely operates within large pre-ribosome particles, e.g., the 90S, and may be involved in early processing of the 90S, thereby primarily affecting 40S subunit synthesis. Not surprisingly, translation-initiation factors such as Tif4631p, Fun12p, Rpg1p, and Eap1p were highly enriched in the polysome fractions (Figure 3A). New1p was also enriched in high-density fractions (Figure 3F).

Several proteins (Jip5p, Ydl063cp, Ydr412wp, Yol022cp, and Yor006cp) shown to cause clear ribosome biogenesis defects following deletion of the gene or depletion of the protein (Figure 2C, 2E) distributed mainly in the low-density fractions (Figure 3A), with Jip5-TAP showing only weak enrichment in the 60S fractions (Figure 3E). This sedimentation behavior could be due to transient interactions of these proteins with pre-ribosomes, or alternatively, loss of these factors could affect ribosome biogenesis indirectly. Another explanation is that the TAP tag might partially disrupt interactions between the ribosome biogenesis factors and the pre-ribosomes. We tested this possibility indirectly by assaying for ribosome biogenesis defects in strains with the TAP-tagged alleles. In several cases, we did observe the tag to confer ribosome biogenesis defects (Bud22-TAP, Bud23-TAP, Ydl063c-TAP, Jip5-TAP, and Bcp1-TAP in Figure 3G–3K). These observations indicate that the TAP tag can compromise the function of proteins, possibly by affecting their interactions with other proteins.

#### Sedimentation patterns measured by quantitative mass spectrometry

**Figure 4:**
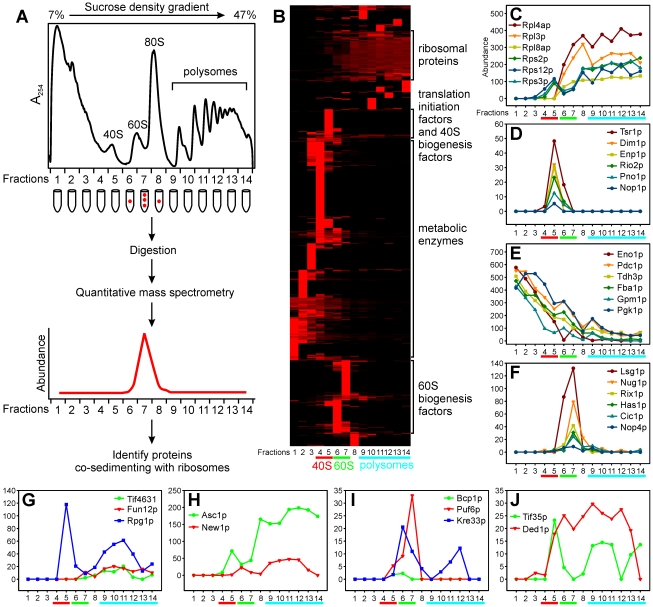
Co-sedimentation of candidate proteins with ribosomal subunits was independently verified using mass spectrometry. (A) Schematic overview of the experimental design. (B) Hierarchical clustering of abundance profiles of 1,023 proteins (row) identified from fractions (columns) of the sucrose density gradient of wild-type yeast cells. Four distinct clusters are enriched (*p*<10^−8^; [83]) for r-proteins, translation-initiation factors and 40S biogenesis factors, metabolic enzymes, and 60S biogenesis factors. Representative profiles are plotted for r-proteins (C), 40S biogenesis factors (D), metabolic enzymes (E), and 60S biogenesis factors (F). (G–J) show profiles for several ribosome biogenesis candidates. Abundance in (C–J) is provided as the frequency of spectral counts (×10,000) of each protein in each fraction; abundance in (B) is further row-normalized.

In order to assay protein co-sedimentation with pre-ribosomes in a tag-independent fashion, we employed a shotgun-style tandem mass-spectrometry (MS/MS) approach [36]. Proteins in each of 14 fractions from a sucrose density gradient separation of the whole-cell lysate from wild-type yeast were identified by mass spectrometry and quantified using MS/MS spectral counts (Figure 4A), which measured the proportion of the total observed MS/MS spectra that were associated with each given protein [37]. Using an approach shown to quantitatively map protein separation profiles in complex samples [38], the distribution of each protein along the density gradient was derived from the normalized abundance profiles obtained across the set of mass-spectrometry analyses (Figure 4A). We identified, on the basis of their sedimentation profiles, a total of 1,023 unique proteins (Table S3) that were clustered into four major groups (Figure 4B; sedimentation profiles of representatives for each group are shown in Figure 4C–4F). Most r-proteins distributed in the high-density fractions corresponding to the polysomes (Figures 4B, 4C), and many translation-initiation factors and 40S biogenesis factors were clustered together and sedimented in the 40S fractions (Figures 4B, 4D). One group primarily distributing in the low-density fractions was highly enriched for metabolic enzymes (Figures 4B, 4E). Finally, many 60S subunit biogenesis factors sedimented in the 60S fractions (Figures 4B, 4F). As controls, the distributions of marker proteins for each of these groups (Eno1p, Tsr1p, Lsg1p, Rps3p, and Rpl8ap) were determined and were found to be consistent with their sedimentation patterns as measured by immunoblot (Figure 3A, Figure 4C–4F), which supports this mass-spectrometry-based approach to measuring the sedimentation pattern for each protein.

Using this approach, we validated several observations from the immunoblots and the known behavior of some of these proteins. Puf6p sedimented in 60S fractions (Figure 4I), while Asc1p and New1p sedimented in the polysome fractions (Figure 4H). The translation-initiation factors Tif4631p, Fun12p, and Rpg1p were enriched in the polysome fractions, which is consistent with their functions (Figure 4G). However, Rpg1p also showed strong enrichment in the 40S fractions in both the tag-based (Figure 3A) and tag-independent (Figure 4G) approaches, which is consistent with its role in eIF3, a complex that associates with free 40S subunits [39]. We also observed sedimentation patterns for Tif35p and Ded1p (Figure 4J), for which TAP-tagged strains were not available. Tif35p is also a component of eIF3 and shows a sedimentation pattern similar to that of Rpg1p (Figure 4G, 4J). The absence of epitope tags in this experiment allowed us to show that Bcp1p was enriched in 60S fractions (Figure 4I) as expected from the 60S biogenesis defect of the *bcp1Δ* mutant (Figure 2C). This was in contrast to the tagged protein, which distributed mainly in the low-density fractions (Figure 3A), consistent with the idea that the tag affects the function of Bcp1p (Figure 3K). Overall, by either the immunoblotting or mass-spectrometry approaches, we could verify that 23 of the candidate proteins co-sedimented with pre-ribosomal subunits.

### Characterization of Genes Affecting Pre-rRNA Processing

**Figure 5:**
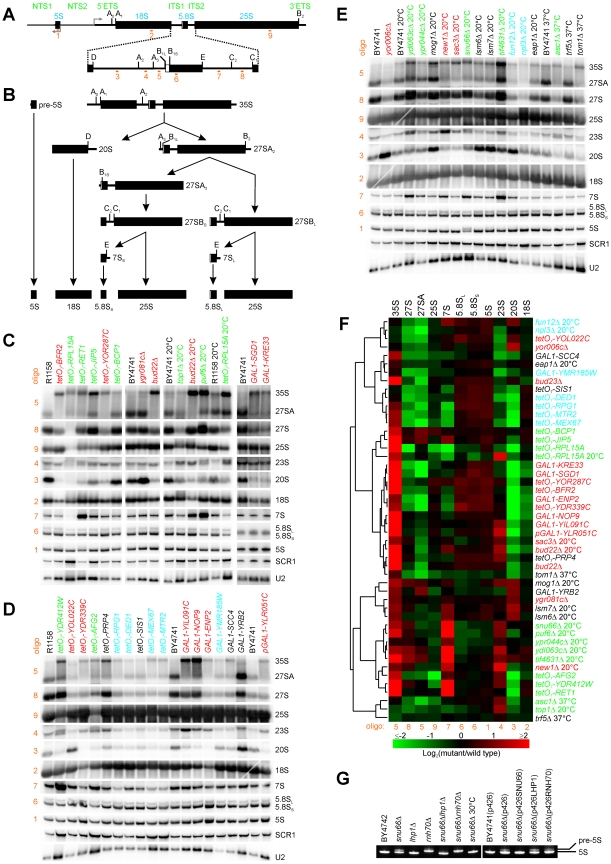
Characterization of mutant pre-rRNA and rRNA processing defects using quantitative Northern blots. (A) Oligonucleotide probes (orange numbers; see Table S2 for sequence information) within an rDNA repeat were selected to probe the majority of pre-rRNA and rRNA species generated during the pre-rRNA processing pathway, diagrammed in (B). Precise processing defects associated with each mutant strain were identified by Northern blots (C–E) of different pre-rRNA and rRNA species in wild-type and mutants. Strain label colors are the same as in Figure 2. (F) Global trends among the mutant strains could be observed from hierarchical clustering of mutant strains on the basis of pre-rRNA and rRNA abundances measured from the Northern blots (C–E), with red and green colors representing increased and decreased levels of RNA species, respectively, in mutants relative to corresponding wild-type control strains. The defect of the *SNU66Δ* strain was examined in more detail in (G). In particular, the temperature-dependent 5S processing defect of this strain could be rescued by deletion of *LHP1*. 5S rRNA was assayed by 10% TBE-Urea gel and SYBR Gold staining. BY4742 is the wild-type control strain for deletion strains, and BY4741(p426) is the wild-type control strain for over-expression strains. Strains were cultured at 20°C unless otherwise indicated.

Most mutants defective for ribosome assembly display altered pre-rRNA processing [9]. The effects on pre-rRNA processing can be a direct consequence of a mutation in an enzymatic processing activity, or they can be indirect. Regardless of whether the effect is direct or indirect, the observed pre-rRNA processing defects provide valuable diagnostics for characterizing the ribosome biogenesis defects and thus the putative activity of a ribosome biogenesis candidate gene; we therefore examined pre-rRNA processing defects in each of the 43 candidate genes confirmed by polysome profiling to affect ribosome biogenesis. Several specific pre-rRNA processing events are critical to biogenesis: The 35S pre-rRNA undergoes extensive modification as well as sequential multiple endo- and exo-nuclease cleavages to give rise to the mature 18S, 5.8S, and 25S rRNAs [2]. The 35S pre-rRNA is first cleaved at sites A_0_, A_1_, and A_2_ to yield 20S and 27SA_2_ species (Figure 5B), and the 20S pre-rRNA is further processed in the cytoplasm to form the mature 18S rRNA after cleavage at the D position. The 27SA_2_ pre-rRNA is processed by two different routes. The majority is cleaved at site A_3_, followed by exonuclease digestion to site B_1S_ to form 27SB_S_, while a small amount of 27SA_2_ undergoes endonucleolytic cleavage at B_1L_ to generate 27SB_L_. Both 27SB species are further processed at sites C_1_ and C_2_ to yield the mature 25S species and 7S species, which mature to 5.8S by 3′-exonuclease digestion to E (Figure 5B).

To examine the detailed effects of the candidate ribosome biogenesis genes on pre-rRNA processing, we used Northern blotting with oligonucleotide probes (Figure 5A) to monitor the levels of 9 different pre-rRNA and rRNA species in each of the 43 mutant strains. In order to quantitatively analyze the change of each RNA species in a mutant relative to the wild-type strain under corresponding conditions, Northern blots (Figure 5C–5E) were quantified, and the logarithm of the intensity ratio of each RNA species from a mutant strain relative to that from its corresponding wild-type strain was calculated and used for hierarchical clustering analysis (Figure 5F). We observed a dramatic increase (red signal in Figure 5F) or decrease (green signal in Figure 5F) of at least one pre-rRNA species for all of the mutants except *eap1Δ* and *trf5Δ* (Figure 5F). The mutants with 60S biogenesis defects in polysome profile analyses clustered into two groups in Northern blotting analyses (Figure 5F, green labels), and many 40S mutants in polysome profile analyses also clustered together (Figure 5F, red labels), showing general correlation between polysome profile defects and pre-rRNA processing defects. In conjunction with the polysome profile and co-sedimentation data, these defects strongly suggest function for the candidate genes in or upstream of the implicated processing steps. We thus employed the observed defects to classify the candidate genes according to their potential general roles.

#### Genes required for processing 35S pre-rRNA

Most mutants displayed increased levels of 35S pre-rRNAs, suggesting direct or indirect roles of the genes in processing A_0_, A_1_, and A_2_ (Figure 5C–5F). One subset of those mutants exhibited reduction of 20S and 18S without affecting the synthesis of 25S, including *GAL1-ENP2*, *tetO_7_-YDR339C*, *GAL1-YLR051C*, *GAL1-NOP9*, *GAL1-YIL091C*, *GAL1-SGD1*, *GAL1-KRE33*, *tetO_7_-YOR287C*, *tetO_7_-BFR2*, and *bud22Δ*, which is consistent with their defects in 40S synthesis observed in polysome profile analyses. Enp2p, Ydr339cp (Fcf1p), Ylr051cp (Fcf2p), Nop9p, Yil091cp, Sgd1p, Kre33p, Bfr2p, and Bud22p localize in the nucleolus [34], consistent with their roles in the early stages of ribosome biogenesis, and with previously known roles for Fcf1p, Fcf2p, Nop9p, and Kre33p in 40S biogenesis [10],[40],[41]. Previous data also suggested that Enp2p and Bfr2p, while nucleolar, are not components of small-subunit processome [42]. Nop9p, Sgd1p, Bfr2p, and Bud22p co-sedimented with 80S/90S fractions (Figure 3A), suggesting direct involvement in 35S processing.

#### Genes involved in 20S pre-rRNA processing

As a second broad classification of pre-rRNA processing defects, we observed accumulation of 20S upon deletion of the genes *YOR006C*, *YGR081C* (*SLX9*), *MOG1*, *FUN12*, *LSM6*, or *LSM7*, or upon depletion of Yol022cp or Yrb2p (Figure 5C–5E), suggesting either defective cleavage at site D or inefficient 43S particle export from the nucleus to the cytoplasm. Note that this is consistent with previous observations for *SLX9* [43], the Lsm complex [44], and *YRB2* [45]. The *yor006cΔ* and *tetO_7_-YOL022C* mutants clustered together in Northern blot analyses (Figure 5F), and no obvious nuclear accumulation of Rps2–green fluorescent protein (GFP) was observed in either mutant (unpublished data), which suggests that these two genes affect pre-rRNA processing at site D. Therefore, *YOR006C* and *YOL022C* were designated as *TSR3* and *TSR4* (Twenty S rRNA accumulation), respectively.

Mog1p has a known role in nuclear protein import [46]. The observed pre-rRNA processing defect may therefore derive from defective nuclear import of ribosome biogenesis factors. Fun12p is a conserved translation-initiation factor that promotes ribosomal subunit joining [47]. Deletion of *FUN12* reduced the levels of 27S and accumulated 20S (Figure 5E). Fun12p also interacts with many ribosome biogenesis factors in large-scale affinity-purification studies [48],[49]. In addition, the polysome profile of *fun12Δ* revealed reduced 40S levels, unlike the profiles for deletion of genes such as *TIF34* and *TIF35* (Figure 2D). Therefore, Fun12p may have a role in processing 20S in the cytoplasm as well as in translation initiation. Lsm6p and Lsm7p are components of the Lsm1p-7p and Lsm2p-8p complexes involved in the mRNA decay and nuclear RNA processing, respectively [50],[51]. Depletion of the essential Lsm2-5 or Lsm8 proteins leads to the delay of pre-rRNA processing and the accumulation of aberrant processing intermediates [44]. We observed that deletion of the nonessential *LSM6* or *LSM7* led to the accumulation of 35S and 20S (Figure 5E), which supports the notion that the Lsm complex affects 20S pre-rRNA processing. Because Yrb2p is involved in export of the ribosome small subunit [45], the accumulation of 20S pre-rRNA in *GAL1-YRB2* likely reflects the accumulation of pre-40S in the nucleus (Figure 5D).

#### Genes required for 27S processing

We also observed a third broad class of mutants with defects in 27S and/or 7S processing, including *tetO_7_-JIP5*, *tetO_7_-BCP1*, *top1Δ*, *ydl063cΔ*, *asc1Δ*, *tetO_7_-YDR412W*, *tetO_7_-AFG2*, *puf6Δ*, and *tif4631Δ*, most of which also accumulated 35S (Figure 5F).

*JIP5*, *BCP1*, *TOP1*, and *YDL063C* largely affected the processing of 35S and/or 27S, whereas *YDR412W*, *PUF6*, and *TIF4631* strongly affected 7S processing, as well as 27S processing (Figure 5C–5E). Depletion of *AFG2* showed a large reduction of both 27S and 25S and a slight increase in 7S (Figure 5D); its role in ribosome biogenesis was recently independently confirmed during the course of our work [52].

#### The intron-encoded U24 snoRNA, but not the coding sequence, of *ASC1* affects 60S biogenesis

**Figure 6:**
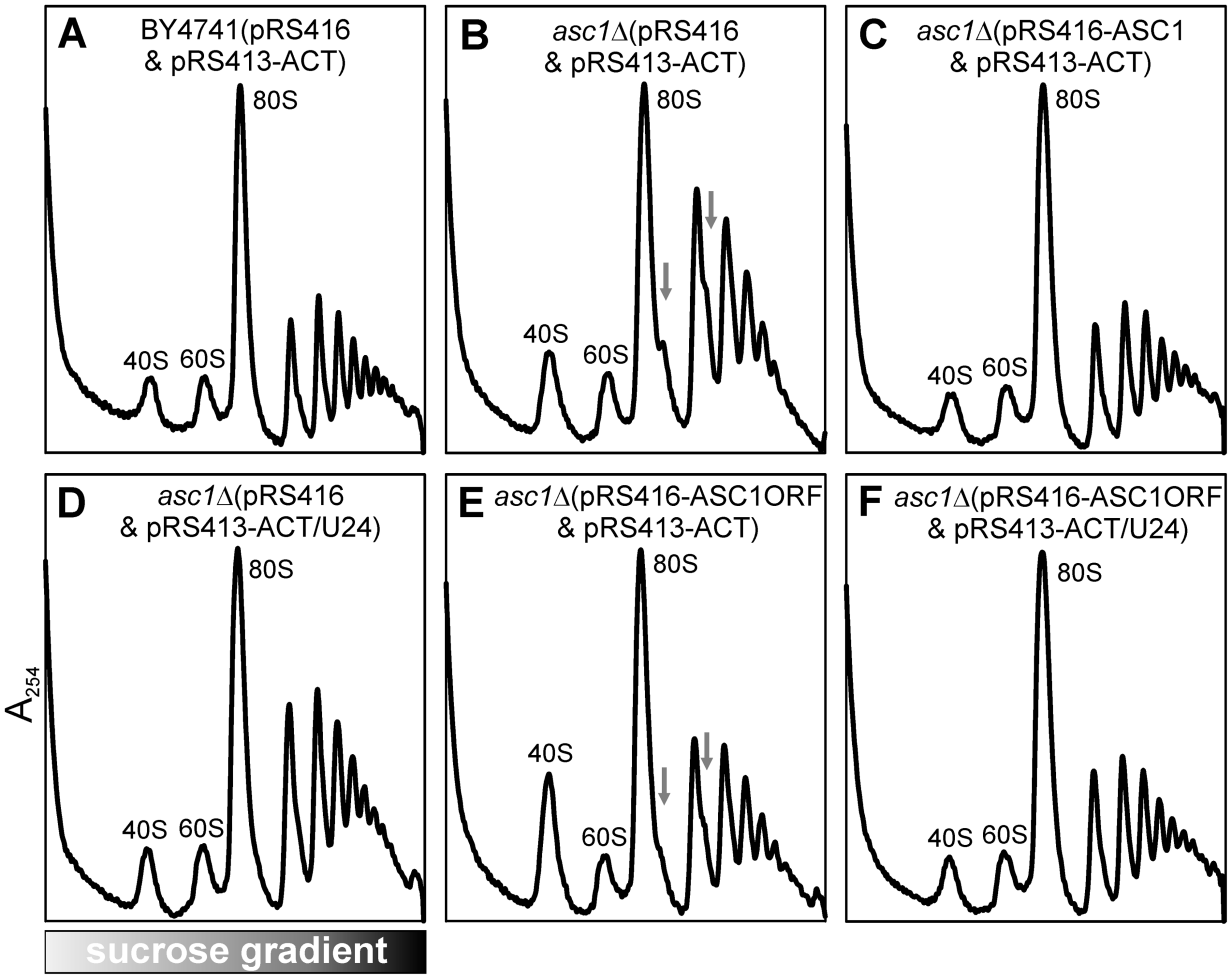
The U24 snoRNA is responsible for the 60S biogenesis defect observed in an *asc1Δ* mutant. Polysome profile of wild-type strain with two control plasmids was shown in (A). The *asc1Δ* mutant with two control plasmids showed the 60S biogenesis defect (B), and this defect was recovered by full length intron-containing *ASC1* gene (C) or intron snoRNA U24 of *ASC1* (D), but not by the coded protein of *ASC1*, deleted of its intron (E). When the intron snoRNA U24 of *ASC1* was put back into the *asc1Δ* mutant expressing the coded protein of *ASC1*, the polysome profile recovered to wild-type (F). All strains were cultured at 37°C. pRS416 and pRS413-ACT are the control plasmids; pRS416-ASC1 carries full length *ASC1* with both intron and exon; pRS413-ACT/U24 carries the intron sequence of *ASC1*; and pRS416-ASC1ORF carries the sequence of protein coding region of *ASC1*. Peaks corresponding to 40S and 60S ribosomal subunits and 80S mono-ribosomes in the polysome profiles were labeled. Gray arrows indicate the halfmer polysomes.

Among the mutants we found to exhibit 27S processing defects, the gene *ASC1* was particularly notable: *ASC1* contains an intron that encodes U24 C/D box small nucleolar RNA required for 2′-O-methylation of 25S at C1437, C1449, and C1450 [53], whereas Asc1 protein has been shown to be a component of the 40S subunit [54]. We observed reductions in 27S, 20S, and 25S upon deletion of both the intron and exons of *ASC1* when cultured at 37°C (Figure 5E), which is consistent with reduced levels of 60S subunits observed in the polysome profile (Figure 2C). In order to determine whether the intron or protein conferred the observed defect, we tested complementation of the *asc1Δ* with each: expression of U24 in *asc1Δ* partially suppressed 60S biogenesis defects observed in the polysome profile analysis, whereas expression of Asc1 protein did not alleviate the defects (Figure 6), which indicates the importance of U24 instead of Asc1p in 60S biogenesis. Ribosomal RNA modifications by snoRNAs have been known for a long time, but their exact physiological roles are generally unclear. Recently, 20 C/D box snoRNAs were shown to phenotypically affect ribosomes [55], and here we demonstrate that rRNA modifications by the intron-encoded snoRNA U24 affect the formation of 60S subunits, demonstrating the importance of an individual snoRNA in ribosome biogenesis.

#### *SNU66* is involved in processing the 5S rRNA precursor

Of all of the 43 mutants tested for rRNA processing defects, only one showed a defect in 5S processing. The 5S rRNA precursor is transcribed by RNA polymerase III and subsequently processed by the 3′ exonuclease Rna82p/Rex1p/Rnh70p (Figure 5B) [56],[57]. In addition to processing defects for 35S, 27S, and 7S upon deletion of *SNU66*, we observed an inefficient processing of the 5S rRNA precursor at 20°C (Figure 5E). Snu66p is a known component of the U4/U6.U5 snRNP complex involved in pre-mRNA splicing [58]. Splicing defects might indirectly affect ribosome biogenesis because 99 out of 137 genes for r-proteins contain introns [59]. However, the unique processing defect for the 5S rRNA precursor was not observed upon depletion of other splicing factors (e.g., *PRP4*, *LSM6*, *LSM7*), suggesting that *SNU66* is involved in both splicing and 5S rRNA biogenesis.

To further elucidate the role of Snu66p in 5S processing, 5S rRNAs from the double-deletion mutants *snu66Δrhn70Δ* and *snu66Δlhp1Δ* were analyzed. As expected, 5S processing was completely blocked upon deletion of both *SNU66* and *RNH70* due to lack of 3′ exonuclease activity conferred by Rnh70p (Figure 5G). However, the 5S precursor was completely processed upon deletion of both *SNU66* and *LHP1* (Figure 5G). Lhp1p is the yeast La protein, and it has been proposed as the chaperone for RNA polymerase III transcripts [60]. Human La protein has been shown to associate with newly synthesized human 5S [61], and recently, Lhp1p was shown to associate with yeast 5S rRNA transcript [62]. Our results demonstrate that the 5S processing defect observed upon deletion of *SNU66* was due to Lhp1p, possibly because it protects the 3′ end of 5S precursor. Accordingly, over-expression of Lhp1p slightly increased the level of 5S precursor in a *snu66Δ* strain (Figure 5G). In addition, the 5S processing defect in *snu66Δ* was not suppressed by over-expressing Rnh70p (Figure 5G), which suggests that Snu66p is required for efficient processing of 5S by Rnh70p at 20°C. Thus, based upon the specificity of the processing defect and genetic interactions with known 5S processing factors, *SNU66* may play a role as a novel 5S processing factor, in addition to its known role in pre-mRNA splicing and its observed defects in 60S biogenesis.

### Identification of New Genes Required for Ribosomal Subunit Export

As a last major characterization of the candidate ribosome biogenesis genes, we investigated their possible roles in ribosome nuclear export. Nuclear export of the ribosomal subunits through NPCs depends upon the RanGTPase cycle and receptor proteins that mediate the interaction between the ribosomal subunit and the NPC. The receptors can bind to adapter proteins or to the subunits directly. In the case of the 60S subunit in yeast, export depends upon the adapter protein Nmd3p and its receptor Crm1p (Xpo1 in human), as well as the heterodimer of Mex67p/Mtr2p [63] and the specialized receptor Arx1p [64],[65]. Export of the 40S subunit also requires Crm1p, and although it has been suggested that Ltv1p acts as a Crm1p-dependent adapter, Ltv1p is not essential, indicating that additional adapters and/or receptors remain to be identified [5],[66]. To test whether the ribosome biogenesis candidates affect ribosome transport, we assayed ribosome export in the mutants by using Rps2-GFP and Rpl25-GFP as reporters for the small and large ribosomal subunits, respectively [10],[67], while monitoring the nucleolus with Sik1-mRFP [34].

**Figure 7:**
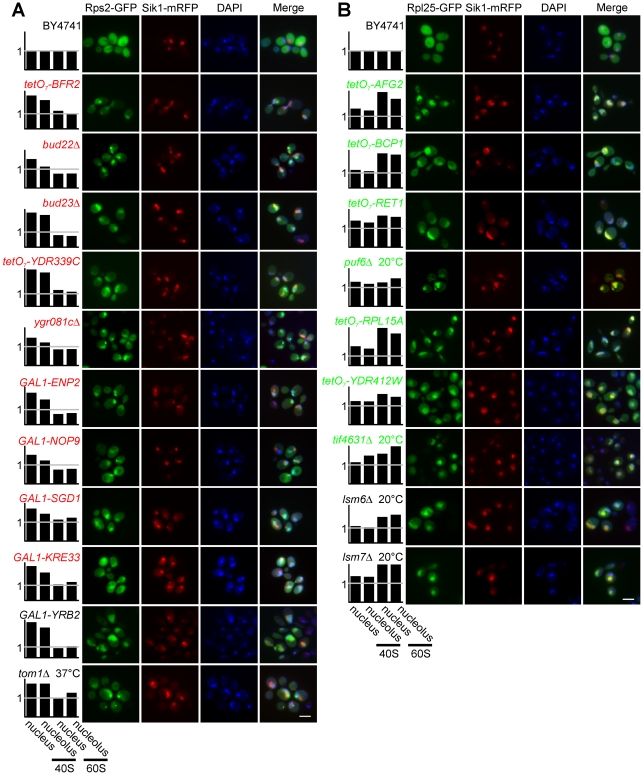
Identification of ribosomal subunit nucleolar and nuclear export defects. (A) Mutants with observed small subunit-export defects. (B) Mutants with large subunit-export defects. The first row of panel (A) and panel (B) represent the wild-type strain BY4741 cultured at 30°C. Rps2-GFP and Rpl25-GFP are reporters for the ribosomal small and large subunits, respectively. Sik1-mRFP is the reporter for the nucleolus. DAPI was used to stain DNA to visualize the nucleus. The white scale bar represents 5 µm. The normalized enrichment of ribosomal subunits in the nucleus or nucleolus relative to the cytoplasm, calculated relative to the appropriate control strain, is plotted for each strain.

In wild-type control strains cultured under various conditions, both small and large ribosomal subunits localized primarily in the cytoplasm (Figure 7A–7B, first row, and Figure S3). Upon depletion of Yrb2p, a known factor involved in small-subunit export [45], ribosomal small subunits accumulated in the nucleus (Figure 7A), while the large subunits were unaffected (Figure S4).

In mutants defective in the synthesis of small subunits, including *tetO_7_-BFR2*, *bud22Δ*, *bud23Δ*, *tetO_7_-YDR339C*, *ygr081cΔ*, *GAL1-ENP2*, *GAL1-NOP9*, *GAL1-SGD1*, and *GAL1-KRE33*, we observed significant accumulation of the small subunit reporter in the nucleus and/or nucleolus (Figure 7A), whereas the large subunits were unaffected (Figure S4). The defective nuclear export of 40S subunits upon depletion of Kre33p is consistent with previous observation of a temperature sensitive mutant *kre33-1* [10]. Because the pre-40S contains the 20S pre-rRNA as it is exported to the cytoplasm, a bona fide block in subunit export is expected to result in increased levels of 20S rRNA. This was in fact observed for the *bud23Δ* and *ygr081cΔ* mutants (Figure 5F), which suggests that they act late in the biogenesis and export pathway, whereas the other genes are involved in early ribosome biogenesis. Recently, Bud23p has also been shown to methylate G1575 of 18S rRNA [68]. We note, however, that defective pre-rRNA processing and/or ribosome assembly may also lead to the inefficient transport of ribosomes to the cytoplasm [69] or accumulation of reporter proteins in the nucleus.

In mutants with defective synthesis of large ribosomal subunits, including *tetO_7_-AFG2*, *tetO_7_-BCP1*, *puf6Δ*, *tetO_7_-YDR412W*, and *tif4631Δ*, strong accumulation of the large ribosomal subunits in the nucleolus and nucleus was observed (Figure 7B), but not of the small subunits (Figure S5). Surprisingly, deletion of *LSM6* or *LSM7* inhibited the transport of pre-60S subunits to the cytoplasm (Figure 7B) but not the small subunits (Figure S5). Therefore, the accumulation of 20S upon deletion of *LSM6* or *LSM7* suggests that they act in 20S processing in the cytoplasm. In total, we identified 17 genes that affected export of either the ribosomal small or large subunits.

## Discussion

### Nonessential Ribosome Biogenesis Genes Frequently Display Conditional or Synthetic Essentiality

As expected, many genes for ribosome biogenesis are essential. However, a large number of nonessential genes are clearly involved in ribosome biogenesis, some of which show strong constitutive or conditional phenotypes (Figure S6). For example, deletion of *PUF6*, *SAC3*, or *SNU66* resulted in strong defects at 20°C but only minor defects at the optimal growth temperature of 30°C. In contrast, the polysome profile of *yor006cΔ* showed 40S biogenesis defects at 30°C but no defects at 20°C. Several nonessential genes, including *YIL096C*, *YCR016W*, *YJL122W*, *YNL022C*, *BUD20*, and *NOP13*, form a tight cluster with known ribosome biogenesis genes in the gene network, and their encoded proteins co-sedimented with either 40S or 60S fractions, supporting them as being components of pre-ribosomes (unpublished data). However, deletion mutants for those genes did not show growth defects at 20°C, 30°C, or 37°C (Figure S1), nor were polysome profiles of the deletion mutants different from wild-type cells (unpublished data).

**Figure 8:**
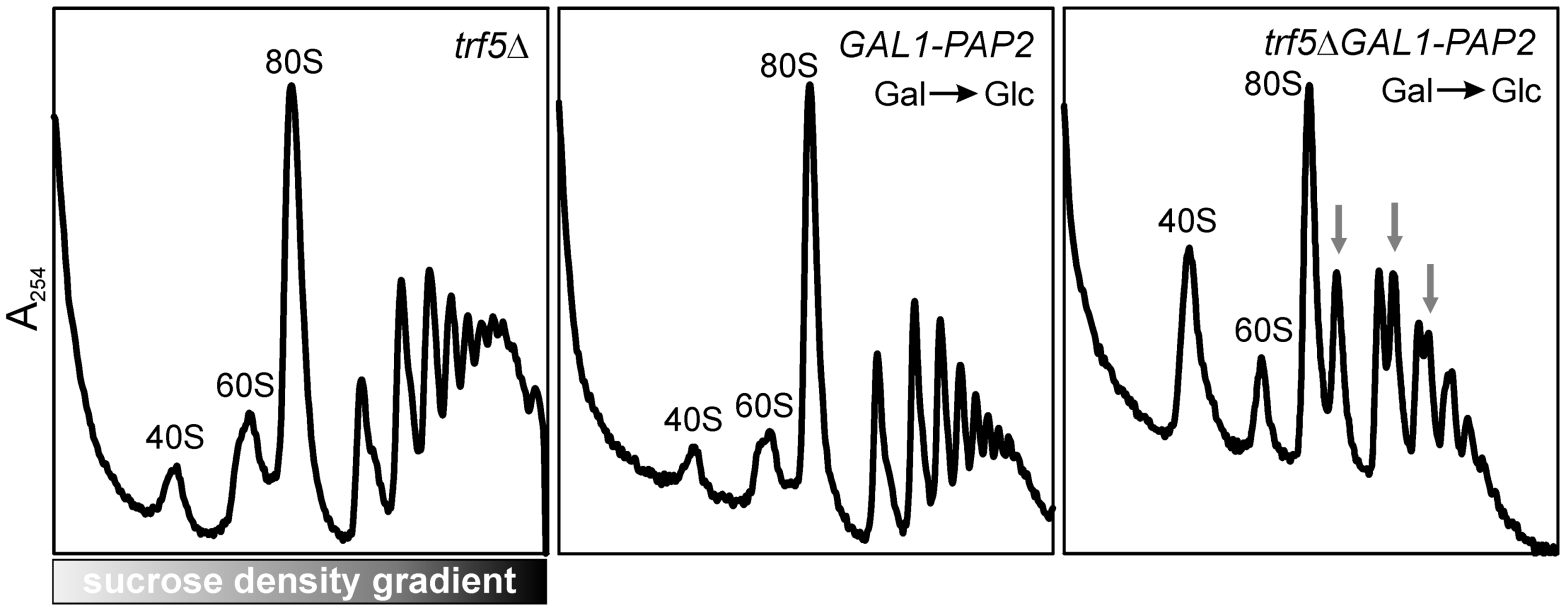
Synthetic ribosome biogenesis defects were observed in a double mutant *trf5ΔGAL1-PAP2*, suggesting that high-scoring genes not confirmed in the previous experiments may often still participate in ribosome biogenesis. The *trf5Δ* mutant was cultured in YPD. *GAL1-PAP2* and *trf5ΔGAL1-PAP2* were first cultured in YPGal, then diluted into YPD and cultured to early logarithmic phase. Gray arrows indicate halfmer polysomes. Strong 60S biogenesis defects were observed for the double mutants, but not in either single gene mutant.

However, lack of a mutant phenotype does not imply that these candidate genes are not part of the ribosome biogenesis pathway. In fact, Yjl122wp (Alb1p) was recently confirmed to interact directly with the known ribosome biogenesis factor Arx1p, although the deletion mutant had no observable phenotype [70]. It is therefore still likely that the remaining candidate genes participate in ribosome biogenesis but that we failed to identify a conditional phenotype or that these genes are functionally redundant with other genes. In the latter case, synthetic interaction assays might prove a useful strategy for deciphering the genes' functions. Indeed, we observed one such example: mutants with either deletion of *TRF5* or depletion of Pap2p did not exhibit defects in polysome profile analyses at 30°C, but depletion of Pap2p in the *trf5Δ* mutant caused strong 60S biogenesis defects evident in polysome profile analysis (Figure 8), which suggests that *TRF5* and its paralog *PAP2* are required for efficient ribosome biogenesis, presumably by facilitating the removal of aberrant pre-rRNA molecules [71]. Thus, many of the remaining nonessential mutants without conditional phenotypes may still be involved in ribosome biogenesis.

### Interactions of Ribosome Biogenesis with mRNA Export and Splicing

Gene network-based predictions based on binary associations between genes intrinsically help to identify genes that participate in multiple cellular processes. Correspondingly, several genes we identified have been reported to have other functions. For example, *BCP1* is required for the export of Mss4p [72], Sgd1p interacts with Plc1p and is involved in osmoregulation [73], and a recent study showed that Mtr2p, known as an mRNA export receptor [74], is directly involved in ribosomal large-subunit export [63]. Similarly, we identified Sac3p, which localized to the NPC and is involved in mRNA export [75] as a ribosome biogenesis factor based on polysome profile and Northern blot analyses of the deletion mutant (Figures 2E, 5E). In addition, Sac3p co-sedimented with 40S fractions, suggesting its possible association with ribosomes (Figure 3A). It is known that Sac3p can mediate protein export [76], but we did not observe export defects for either ribosomal subunit in the *sac3Δ* mutant (unpublished data). Thus, *SAC3* joins *MTR2* and *MEX67* as genes participating in both the ribosome biogenesis and mRNA export pathways.

Recently, the splicing factor Prp43p was confirmed to be a ribosome biogenesis factor by several groups, which suggests coordination of ribosome biogenesis and mRNA splicing [77]–[79]. We observed that four genes associated with mRNA splicing—*LSM6*, *LSM7*, *PRP4*, and *SNU66*—also play roles in ribosome biogenesis. Although we do not exclude the possibility of indirect roles of *PRP4* in ribosome biogenesis, deletion of *SNU66* (a component of the tri-snRNP) not only delays 35S processing but also affects processing of the 5S rRNA precursor (Figure 5E). Thus, these data provide further evidence for shared components between these processes, which supports a general connection between ribosome biogenesis and mRNA splicing. Whether this connection is direct or indirect generally remains to be established, although the specificity of the rRNA processing defect and the observed genetic interactions (Figure 5G) suggest a direct role for *SNU66* in 5S processing.

### Conclusions

In conclusion, we applied the emerging technique of network-guided genetics to computationally predict and experimentally validate at least 15 previously unreported ribosome biogenesis genes (*TIF4631*, *SNU66*, *YDL063C*, *JIP5*, *TOP1*, *SGD1*, *BCP1*, *YOR287C*, *BUD22*, *YIL091C*, *YOR006C/TSR3*, *YOL022C/TSR4*, *SAC3*, *NEW1*, *FUN12*) (Table 1), most of which have human orthologs and thus represent evolutionarily conserved components of this essential core cellular process. Selecting candidates with a network-guided genetics approach therefore proved to be a powerful approach for identifying new genes in a pathway, even in such a well-studied cellular process as ribosome biogenesis, with ∼40% of the tested genes in the polysome profile analyses being shown to participate in this pathway. Although considerable effort has been spent predicting and validating gene functions from diverse functional genomics and proteomics data [17],[80], to our knowledge this is one of the most extensive experimental tests of predictions from network-guided genetics. These results add >10% new genes to the ribosome biogenesis pathway, significantly extending our understanding of a universally conserved eukaryotic process.

**Table 1:** Summary of the evidence from this study for the involvement of known and new proteins in ribosome biogenesis.

ORF	Gene^a^	Human Ortholog^b^	Number of Links to Seed Genes	Network Evidence^c^	Mutant Growth	Polysome Profile Defect	Co-sedimentation^d^	Pre-rRNA Processing Defect	Ribosome Export Defect
*YGR162W*	*TIF4631*	*EIF4G1*, *EIF4G*, *EIF4G3*	22	MS, CX, LC	Slow	60S	Across gradient	35S, 27S, 7S, 20S	60S
*YOR308C*	*SNU66*	*SART1*	8	MS, CC, LC	Slow at 20°C	60S	40S	35S, 27S, 5S	No
*YDL063C*	—	—	5	MS, CC, YH, CX	Slow	60S	Free	35S, 27S	No
*YDR412W*	*RRP17*	?*NOL12*	14	CX, MS, YH	Essential	60S	Free	35S, 7S	60S
*YPR169W*	*JIP5*	?*AAC69625*	19	CX, MS	Essential	60S	Free, 60S	35S, 27S	No
*YOL006C*	*TOP1*	*TOP1*	7	CC, MS, LC, CX	Slow	60S	Across gradient	35S, 27S	No
*YNL132W*	*KRE33* [10]	*NAT10*	77	MS, CX, LC	Essential	40S	—	35S	40S
*YDR496C*	*PUF6* [21]	*KIAA0020*	94	CX, MS, LC	Slow at 20°C	60S	60S	35S, 27S, 7S	60S
*YLR336C*	*SGD1*	*NOM1*	31	CX, MS	Essential	40S	40S, 60S, 80S	35S	40S
*YLR397C*	*AFG2* [52]	*SPATA5*	7	CX, MS, CC	Essential	60S	—	35S, 7S	60S
*YDR361C*	*BCP1*	*BCCIP*	19	CX	Essential	60S	Free, 60S	35S	60S
*YJL010C*	*NOP9* [40]	*C14orf21*	56	CX, LC	Essential	40S	40S, Polysome	35S	40S
*YOR287C*	—	*C6orf153*	40	CX, MS	Essential	40S	—	35S	No
*YDR339C*	*FCF1* [41]	*CN111_HUMAN*	13	CX	Essential	40S	—	35S	40S
*YMR014W*	*BUD22*	—	37	CX, MS	Slow	40S	80/90S, Polysome	35S	40S
*YCR047C*	*BUD23* [68]	*WBSCR22*	7	MS, CX	Slow	40S	40S	35S, 20S	40S
*YLR051C*	*FCF2* [41]	*DNTTIP2*	13	CX	Essential	40S	—	35S	—
*YGR145W*	*ENP2*	*NOL10*	91	CX, MS, LC, RS	Essential	40S	—	35S	40S
*YDR299W*	*BFR2*	*AATF*	71	CX, MS, LC	Essential	40S	40S, 80/90S	35S	40S
*YIL091C*	—	*DEF*	12	CX, MS	Essential	40S	40S	35S	No
*YOL022C*	*TSR4*	?*PDCD2L*	30	CX	Essential	40S	Free	20S	No
*YOR006C*	*TSR3*	*C16orf42*	2	CX	Slow at 20°C and 30°C	40S	Free	20S	No
*YGR081C*	*SLX9* [43]	—	14	MS, CX, GT	Slow at 30°C	40S	40S	20S	40S
*YDR159W*	*SAC3*	*MCM3AP*	1	LC	Slow	40S	40S, 80/90S	35S	No
*YPL226W*	*NEW1*	?*ABCF1*	8	CX, MS	Slow at 20°C and 30°C	40S	Across gradient	35S	No
*YJR074W*	*MOG1*	*RANGRF*	3	CC, GT, MS, LC, YH	Slow	Minor	Free	35S, 27S, 20S	No
*YAL035W*	*FUN12*	*EIF5B*	40	MS, GN, CX	Slow	40S	Polysome	20S	No
*YPR178W*	*PRP4*	*PRPF4*	11	MS, LC, CC, YH	Essential	Minor	Free, 40S	35S	No
*YDR378C*	*LSM6* [44]	*LSM6*	7	MS, LC, CC, YH, TS	Slow	Minor	—	35S, 20S	50% cells 60S
*YNL147W*	*LSM7* [44]	*LSM7*	7	MS, LC, CC, YH, TS	Slow	Minor	Polysome	35S, 20S	50% cells 60S

a Citations indicate prior evidence for roles in ribosome biogenesis. Note that all genes listed have at least some prior evidence (e.g., protein interactions, expression patterns, or localization, as indicated in the network evidence column), as this forms the basis of the computational predictions; only studies reporting detailed characterization are indicated here.

b Human orthologs were identified using INPARANOID. For genes without clear orthologs, the best BLASTP hits are indicated by a question mark (?).

c CC, co-citation; CX, co-expression; GN, gene neighbor; GT, genetic interaction; LC, literature-curated protein-protein interaction; MS, mass spectrometry analysis of purified complex; PG, phylogenetic profile; RS, Rosetta Stone protein (gene fusion); TS, protein tertiary structure inferred protein-protein interaction; YH, high-throughput yeast two hybrid.

d Free represents the low density fractions.

## Materials and Methods

### Strains

Haploid MATa deletion mutants [81] were obtained from Research Genetics. TetO_7_-promoter mutants [28] and TAP-tagged strains [31] were acquired from Open Biosystems. All commercial strains in this paper were verified by PCR, and four strains found to be incorrect in commercial collections (*ypr045cΔ*, *tetO_7_-SGD1*, Kre33-TAP, and *tetO_7_-KRE33*) were recreated. GAL1-promoter mutants were constructed in strain BY4741 (Text S1).

Haploid deletion mutants were cultured to OD_600_ 0.3–0.5 in YPD (1% yeast extract, 2% peptone, 2% dextrose) at the conditional temperature (20°C, 30°C, or 37°C). TetO_7_-promoter mutants were cultured in YPD and then diluted into YPD with 10 ug/ml doxycycline (Fisher Scientific) for 9–20 h to OD_600_ 0.3–0.5. GAL1-promoter mutants were cultured in YPGal (1% yeast extract, 2% peptone, 2% galactose) and then diluted into YPD for 12–20 h to OD_600_ 0.3–0.5. Strains carrying pRS416 and pRS413 derived plasmids were cultured in synthetic complete media minus uracil and histidine supplemented with 2% dextrose to OD_600_ 0.3–0.5. Detailed culture information for each individual strain is described in Table S1.

### Polysome Profile Analyses

Yeast cells were cultured at various conditions to OD_600_ 0.3–0.5. Two hundred µg/ml cycloheximide (Sigma) was added to each culture. Cell lysate preparation and sucrose density gradient sedimentation were performed as previously described (Text S1) [65]. Each mutant's polysome profile was aligned to the wild-type reference polysome profile using COW implemented in MATLAB [29]. Aligned polysome profiles were hierarchically clustered using Cluster and Treeview software [30].

### Immunoblot Analyses

TAP-tagged strains were cultured in YPD at 30°C to OD_600_ 0.3–0.5, and subsequent steps were performed in the same manner as for the polysome profile analyses. Fractions from the sucrose density gradient were collected, and 25 µl of each fraction was deposited onto a nitrocellulose membrane using a 96-well dot-blot system (Schleicher & Schuell). The membrane was probed for the TAP-tagged proteins with the rabbit peroxidase anti-peroxidase soluble complex (Rockland Immunochemicals), using Luminol (Santa Cruz Biotechnology) as the substrate for detection. The total intensity of each dot was quantified with Quantity One 1-D Analysis software (Bio-Rad).

### Mass Spectrometry

The wild-type strain BY4741 was cultured in YPD at 30°C to OD_600_ 0.3–0.5 and then lysed and fractionated on a sucrose density gradient in the same manner as for the polysome profile analyses. Proteins from each fraction were precipitated with 10% cold trichloroacetic acid, washed with cold 100% acetone, resuspended in 100 mM Tris buffer (pH 8.0), and digested with proteomic-grade trypsin (Sigma) for 24 h at 37°C. Each digested peptide mixture was separated by a strong cation-exchange column, followed by a reverse-phase C18 column. Peptides were analyzed online with an electrospray ionization ion-trap mass spectrometer (ThermoFinnigan DecaXPplus), and proteins were identified at a 5% false-detection rate by using PeptideProphet and ProteinProphet software [82]. For each sucrose gradient fraction, the number of MS/MS spectra associated with a given protein was divided by the sum of the spectral counts across all proteins in that fraction to estimate the relative abundance of each protein within each fraction. The resulting relative abundance profiles were subjected to hierarchical clustering using the Cluster and Treeview programs. Raw mass-spectrometry data are deposited in the Open Proteomics Database as accession opd00106_YEAST.

### Northern Blot Analyses

RNA was extracted by the hot acidic phenol method. The high- and low-molecular-weight RNA species were separated by 1% agarose-formaldehyde gel (NorthernMax, Ambion) and 8% polyacrylamide-TBE-urea gel, respectively. RNAs were transferred onto Zeta-Probe GT membrane (Bio-Rad) by capillary transfer for agarose gel or semi-dry electroblotting for polyacrylamide gel. After UV cross-linking of the RNAs to the membrane, 5′-P^32^-labeled oligonucleotide probes were sequentially hybridized, and the hybridization signals were detected by phosphorimaging and quantified using Quantity One (Bio-Rad). The logarithm ratio of total intensity of each RNA species from a mutant to that from the corresponding wild-type was calculated and used for hierarchical clustering.

### Ribosomal Subunit Export Assay

Wild-type strains or mutants were transformed with either pAJ907 (*RPL25-GFP CEN LEU2*) or pAJ1486 (*RPS2-GFP CEN LEU2*), and each strain was also transformed with pRS411-SIK1-mRFP (*SIK1-mRFP CEN MET15*). Strains were cultured in synthetic complete media minus leucine and methionine, supplemented with 2% dextrose or 2% galactose. Essential gene expression was inactivated in the same way as for the polysome profile analyses. Cells were fixed with 4% formaldehyde (Pierce) for 30 min and then washed twice with PBS (pH 7.2). DAPI (Vector Laboratories) was used to stain DNA, and images were acquired using a Nikon E800 microscope and a Photometrics CoolSNAP ES CCD camera. The GFP median intensities within the three different compartments (cytoplasm, nucleus, and nucleolus) for each cell were determined by custom image-processing software implemented in MATLAB (Text S1). Then the relative ratio of GFP median intensity in the nucleus or nucleolus to that in the cytoplasm for each cell was calculated. For each strain, the median of this ratio for a population of cells was used as an index for the enrichment of ribosomal subunits in either the nucleus or nucleolus. To compare this enrichment in mutants to that in their corresponding wild-type strains, the index of each strain was normalized to the index of the corresponding wild-type strain.

## Supporting Information

Figure S1 **Growth assay for nonessential gene deletion mutants.** Deletion mutants were cultured in YPD and diluted to OD_600_ 0.1. A 5-fold series of dilutions were made for each mutant and 5 µl diluted sample was deposited onto a YPD plate. Mutants were cultured at three different temperature conditions (20°C, 30°C, and 37°C). The mutants with slow growth phenotypes in any one of the conditions were highlighted in gray.
(6.72 MB PDF)

Figure S2 **Polysomal profiles of mutants with slightly imbalanced ribosomal subunits.** Mutants were cultured at 30°C unless otherwise indicated in the figure. Peaks corresponding to 40S and 60S ribosomal subunits and 80S mono-ribosomes in the polysome profiles are labeled.
(0.20 MB TIF)

Figure S3 **Ribosomal subunit nuclear export assay in wild-type yeast strains under different conditions.** (A) Ribosomal small subunits mainly localize to the cytoplasm of wild-type strains under assayed conditions. Rps2-GFP and Sik1-mRFP were used as the reporters for 40S small subunits and the nucleolus, respectively. DAPI was used to stain the nucleus. BY4741 is the control strain for the deletion mutants and the strains with GAL1-promoter controlled alleles. R1158 is the control strain for the strains with tetO_7_-promoter controlled alleles. The strains were cultured at 30°C unless otherwise indicated in the figure. The white scale bar at the bottom-right corner represents 5 µm. (B) Ribosomal large subunits mainly localize to the cytoplasm of wild-type strains under assayed conditions. Rpl25-GFP was used as the reporter for 60S large subunits.
(1.63 MB TIF)

Figure S4 **Ribosomal 60S subunit nuclear export was largely unaffected in mutants with 40S nuclear export defects (** **Figure 7A** **).** BY4741 is the representative control strain. Strains with GAL1-promoter controlled alleles or tetO_7_-promoter controlled alleles were cultured as described in Text S1. Labels in this figure conform to Figure S3.
(2.04 MB TIF)

Figure S5 **Ribosomal 40S subunit nuclear export was largely unaffected in mutants with 60S nuclear export defects (** **Figure 7B** **).** BY4741 is the representative control strain. Strains with GAL1-promoter controlled alleles or tetO_7_-promoter controlled alleles were cultured as described in Text S1. Labels in this figure conform to Figure S3.
(1.65 MB TIF)

Figure S6 **Polysomal profiles of mutants cultured at different temperatures.** Strains were cultured at 20°C, 30°C, and 37°C. Peaks corresponding to 40S and 60S ribosomal subunits and 80S mono-ribosomes in the polysome profiles were labeled. Gray arrows indicate the halfmer polysomes. Different mutants showed different temperature-dependent defects in the synthesis of ribosomal subunits.
(0.35 MB TIF)

Table S1 **Ribosome biogenesis candidate genes.** Deletion and conditionally essential strains used in this study and detailed culture conditions for mutants showing defects in polysome profile analyses.
(0.04 MB XLS)

Table S2 **Oligonucleotides used in this study.** Nucleic acid sequences for probes used in Northern blots, primers used for GAL1 promoter tagging, and primers used for cloning.
(0.02 MB XLS)

Table S3 **Protein sedimentation patterns by mass spectrometry.** Sucrose density fractions were analyzed by quantitative mass spectrometry. Each protein's relative abundance is represented by the normalized spectral frequency per fraction.
(0.36 MB XLS)

Text S1 **Supplemental methods and references.**
(0.06 MB DOC)

## Abbreviations

GFP: green fluorescent protein
MS/MS: tandem mass-spectrometry
NPC: nuclear pore complex
rDNA: ribosomal deoxyribonucleic acid
r-protein: ribosomal protein
rRNA: ribosomal ribonucleic acid
pre-rRNA: precursor ribosomal ribonucleic acid
TAP: tandem-affinity purification
