# Supplementary figures and images for: Rational Extension of the Ribosome Biogenesis Pathway Using Network-Guided Genetics
Source: PLoS Biol. 2009 Oct 6;7(10):e1000213. doi: 10.1371/journal.pbio.1000213 (PMC2749941; doi:10.1371/journal.pbio.1000213)

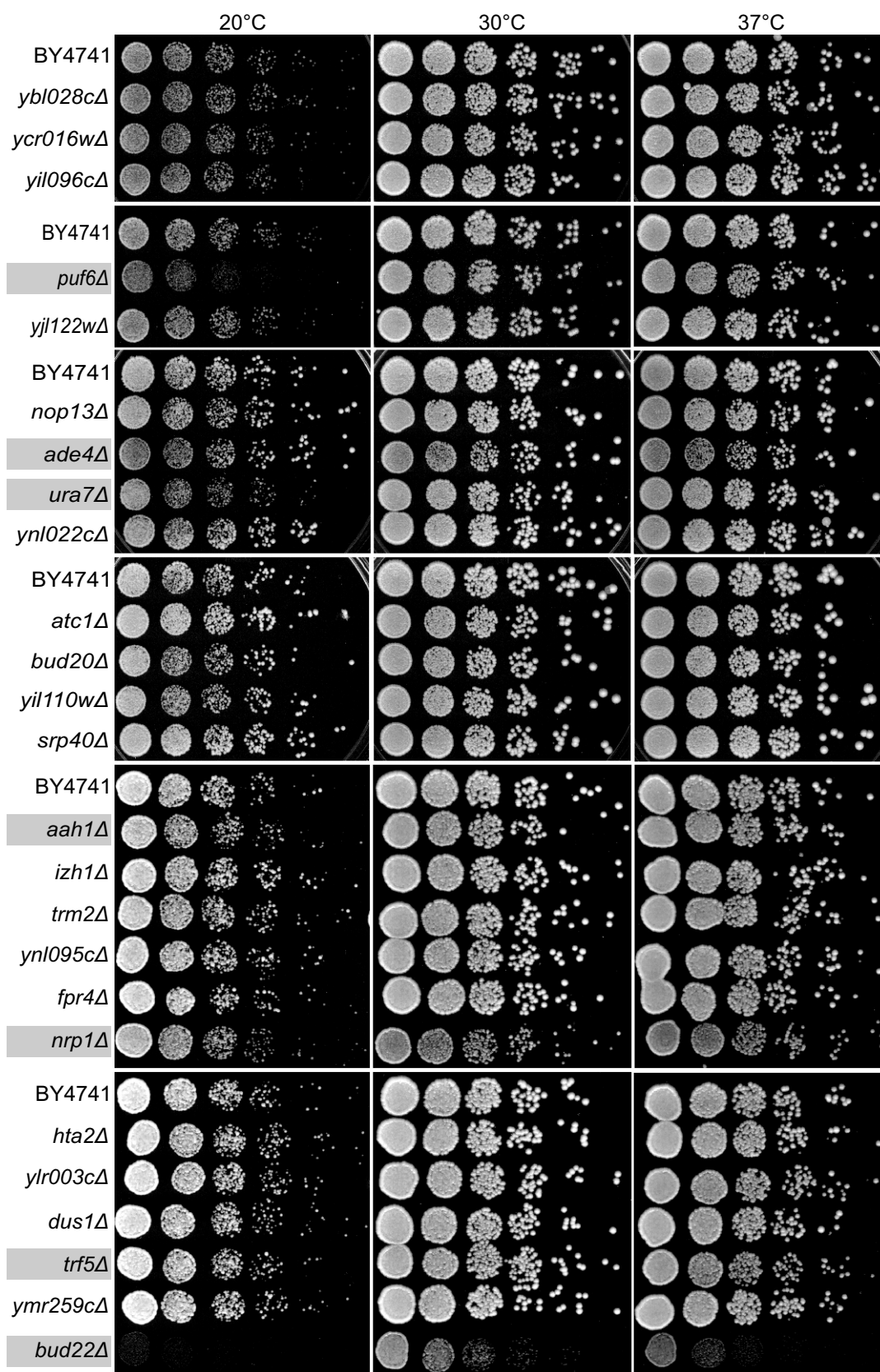

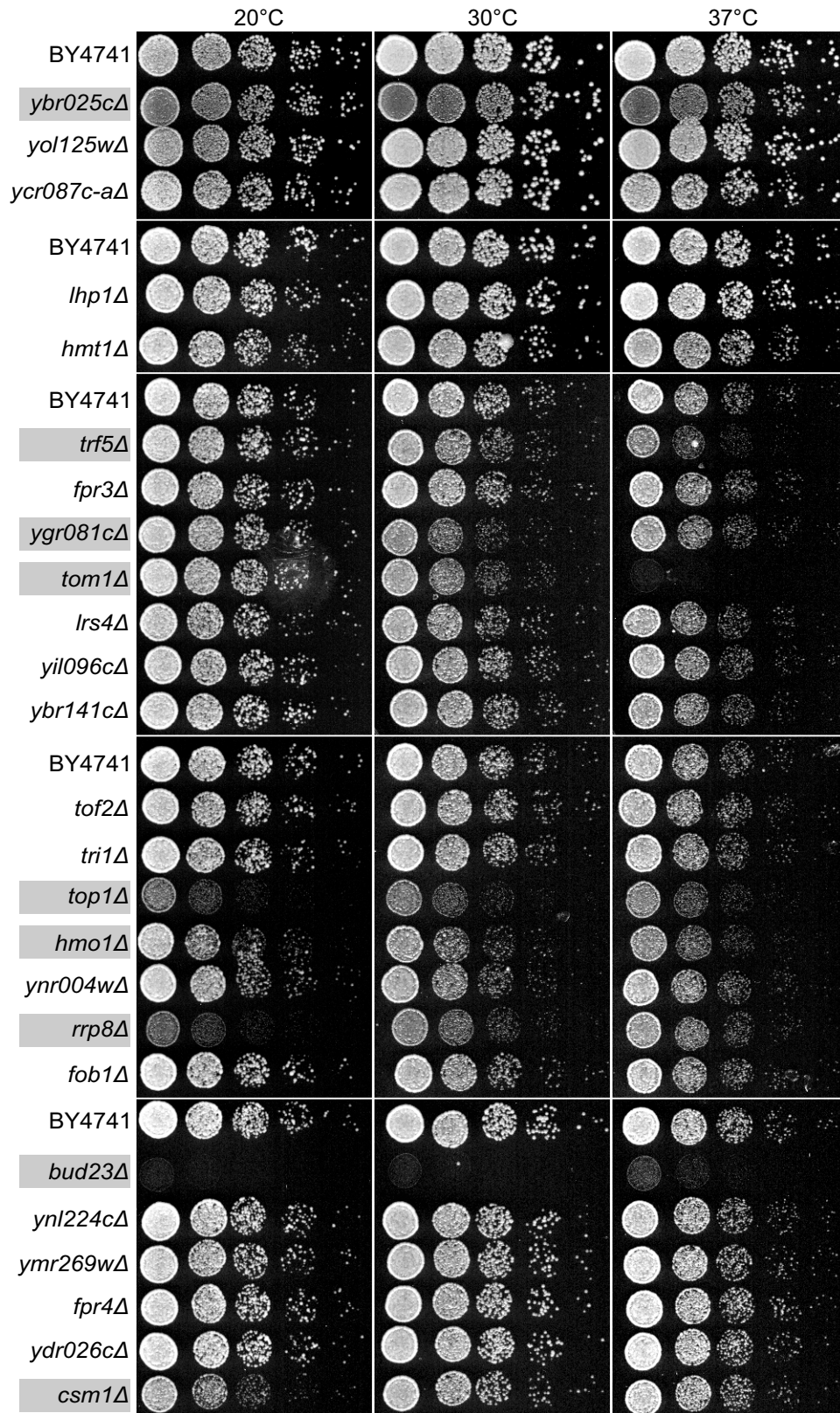

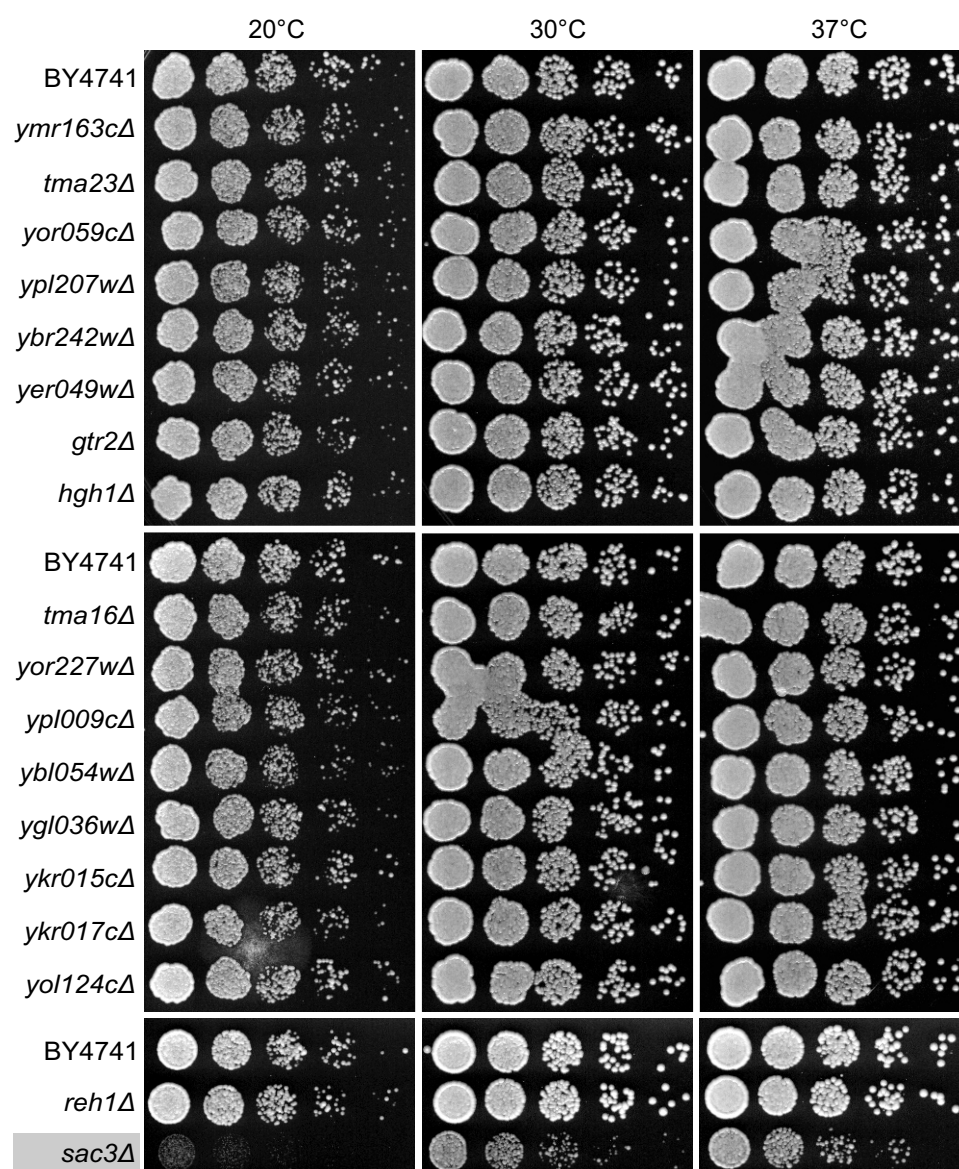

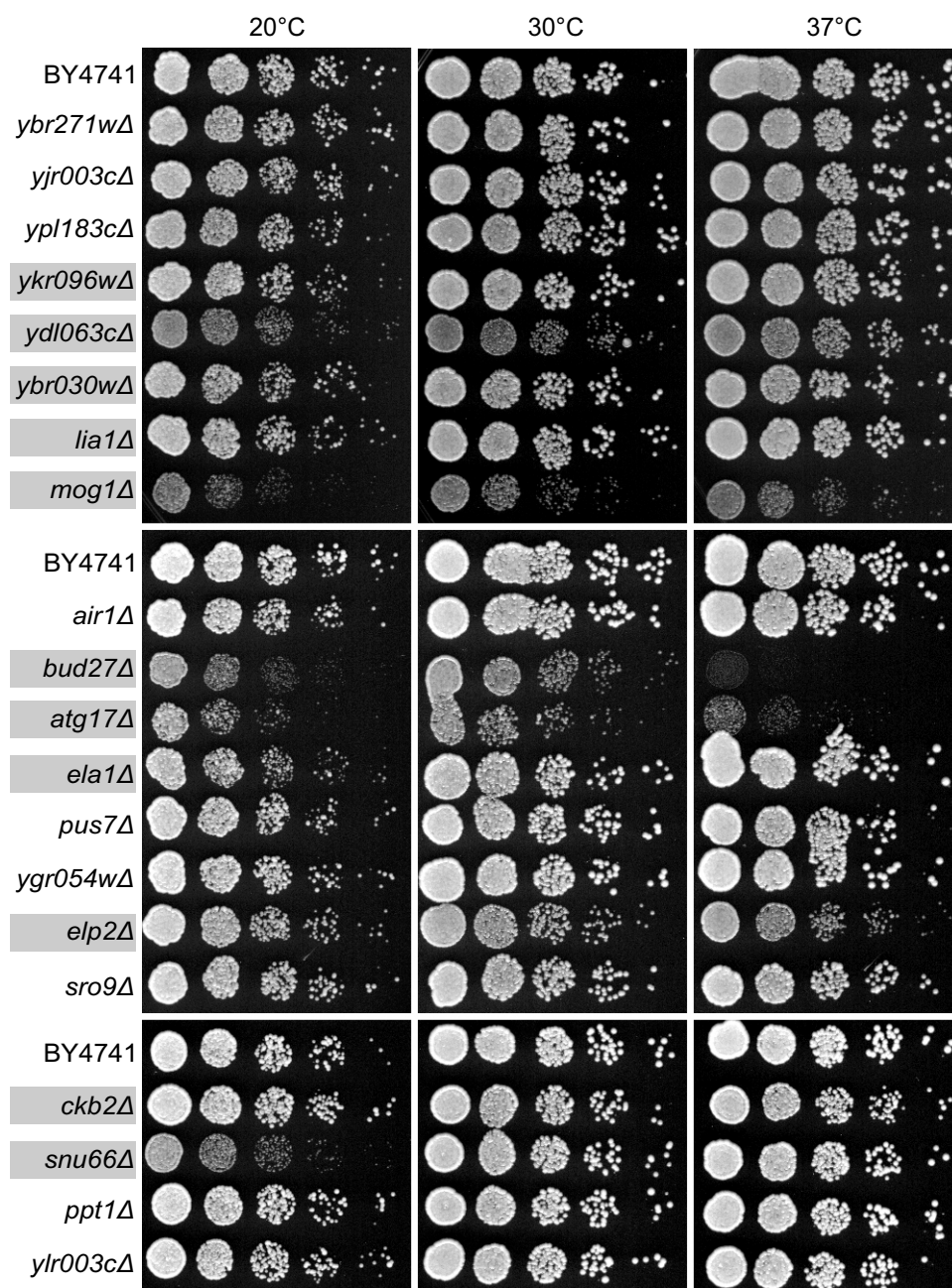

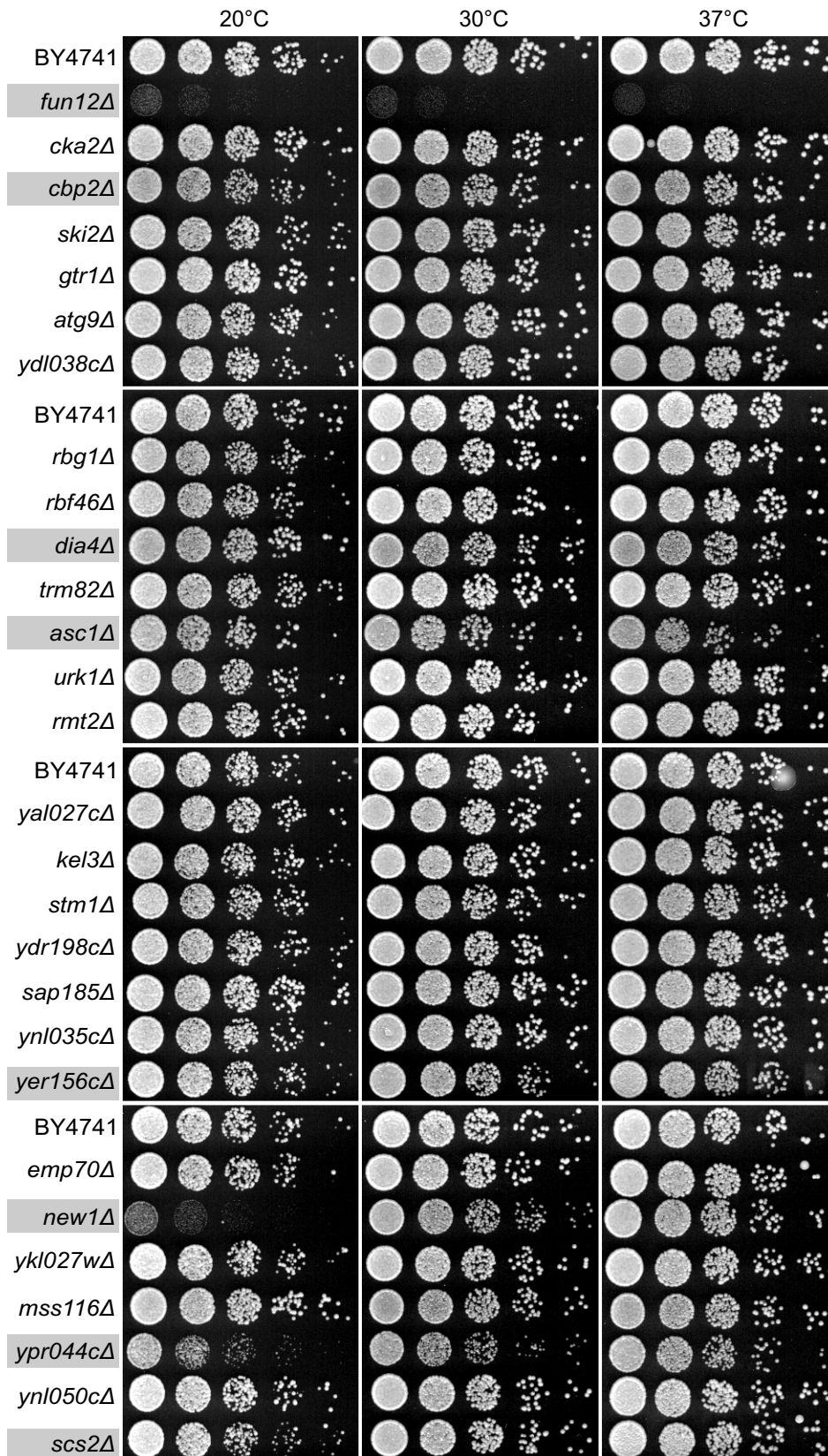

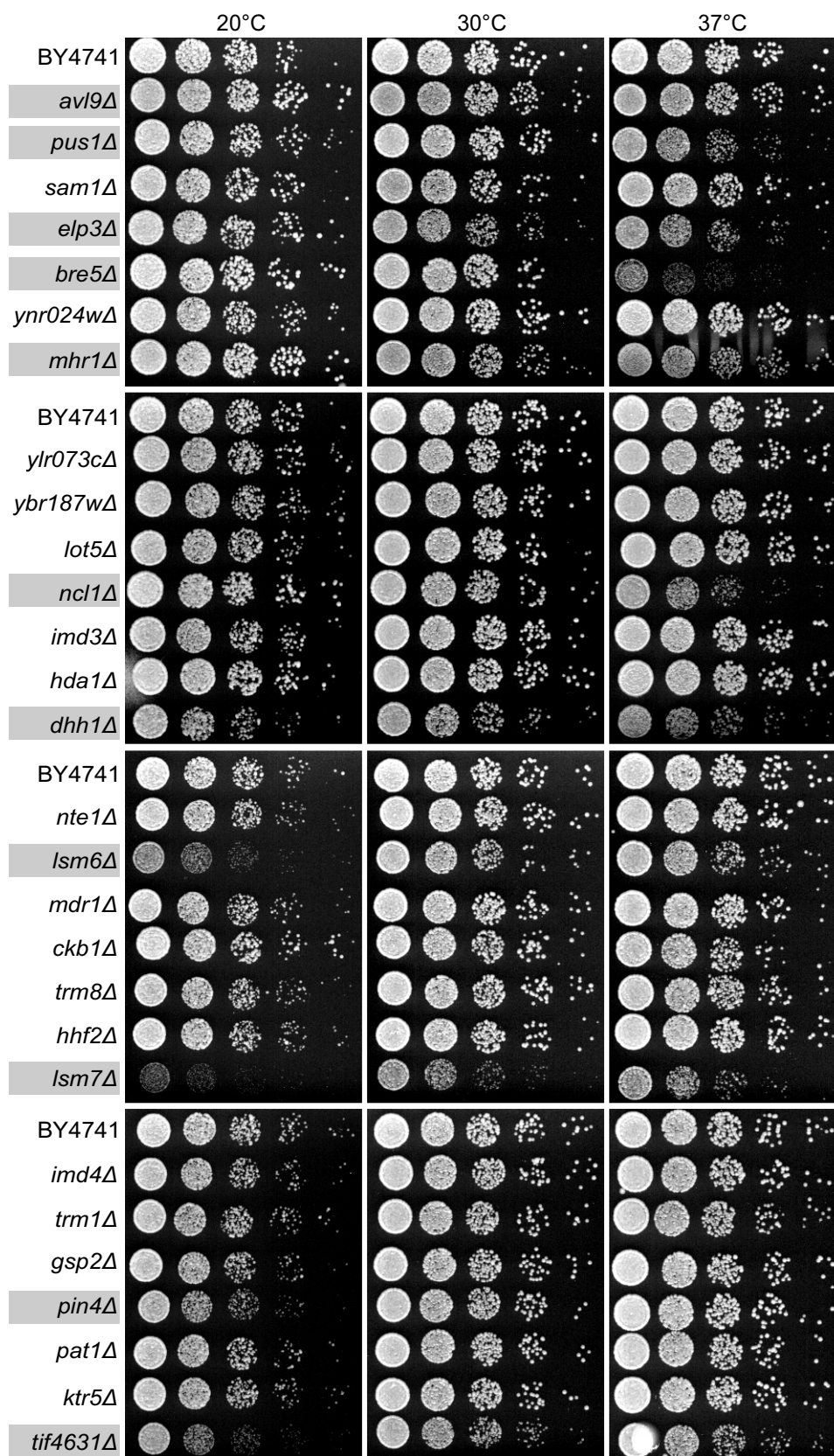

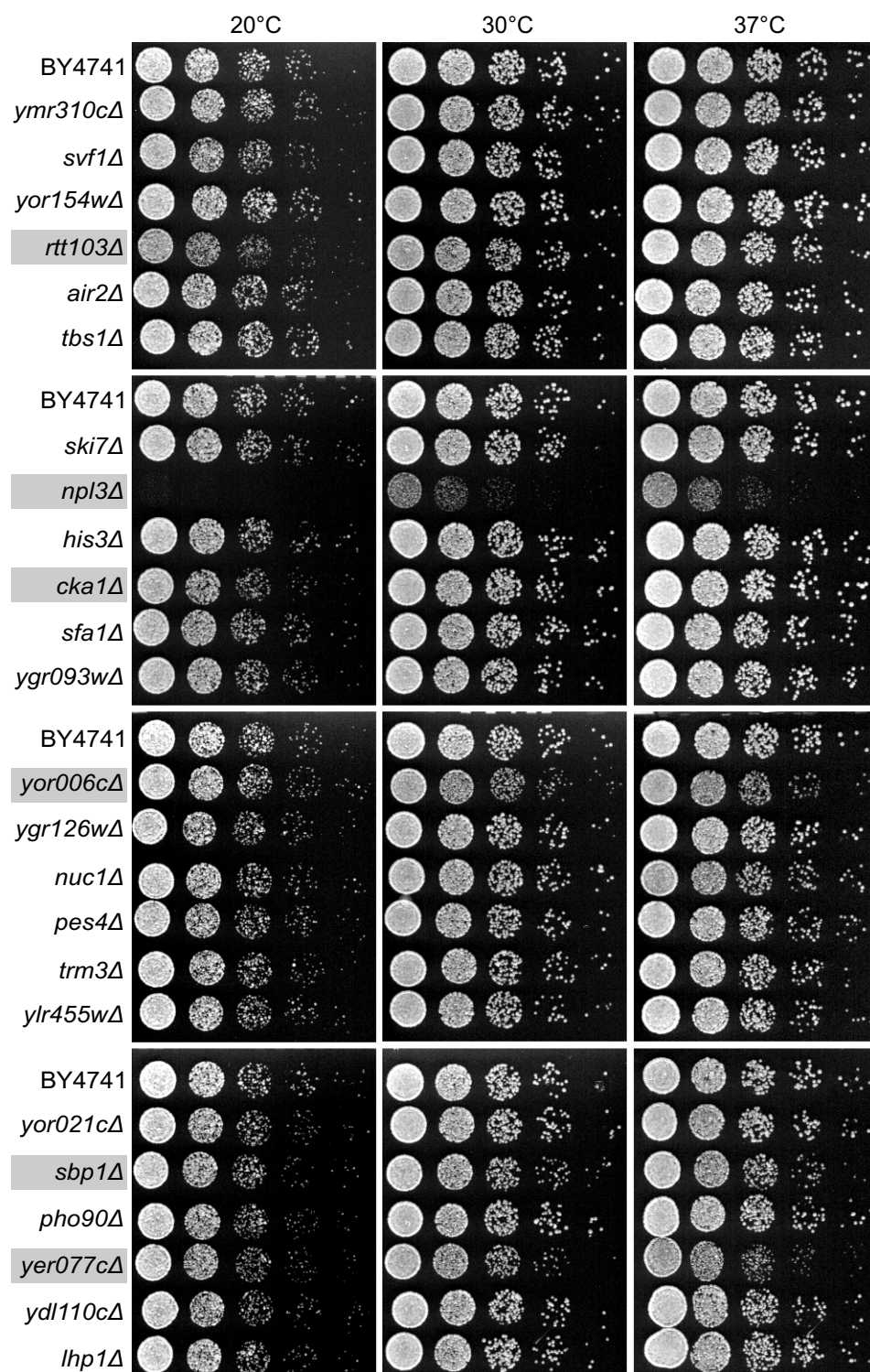

Supplement: Figure S1 — Growth assay for nonessential gene deletion mutants. Deletion mutants were cultured in YPD and diluted to OD600 0.1. A 5-fold series of dilutions were made for each mutant and 5 µl diluted sample was deposited onto a YPD plate. Mutants were cultured at three different temperature conditions (20°C, 30°C, and 37°C). The mutants with slow growth phenotypes in any one of the conditions were highlighted in gray. (6.72 MB PDF) [file pbio.1000213.s001.pdf]

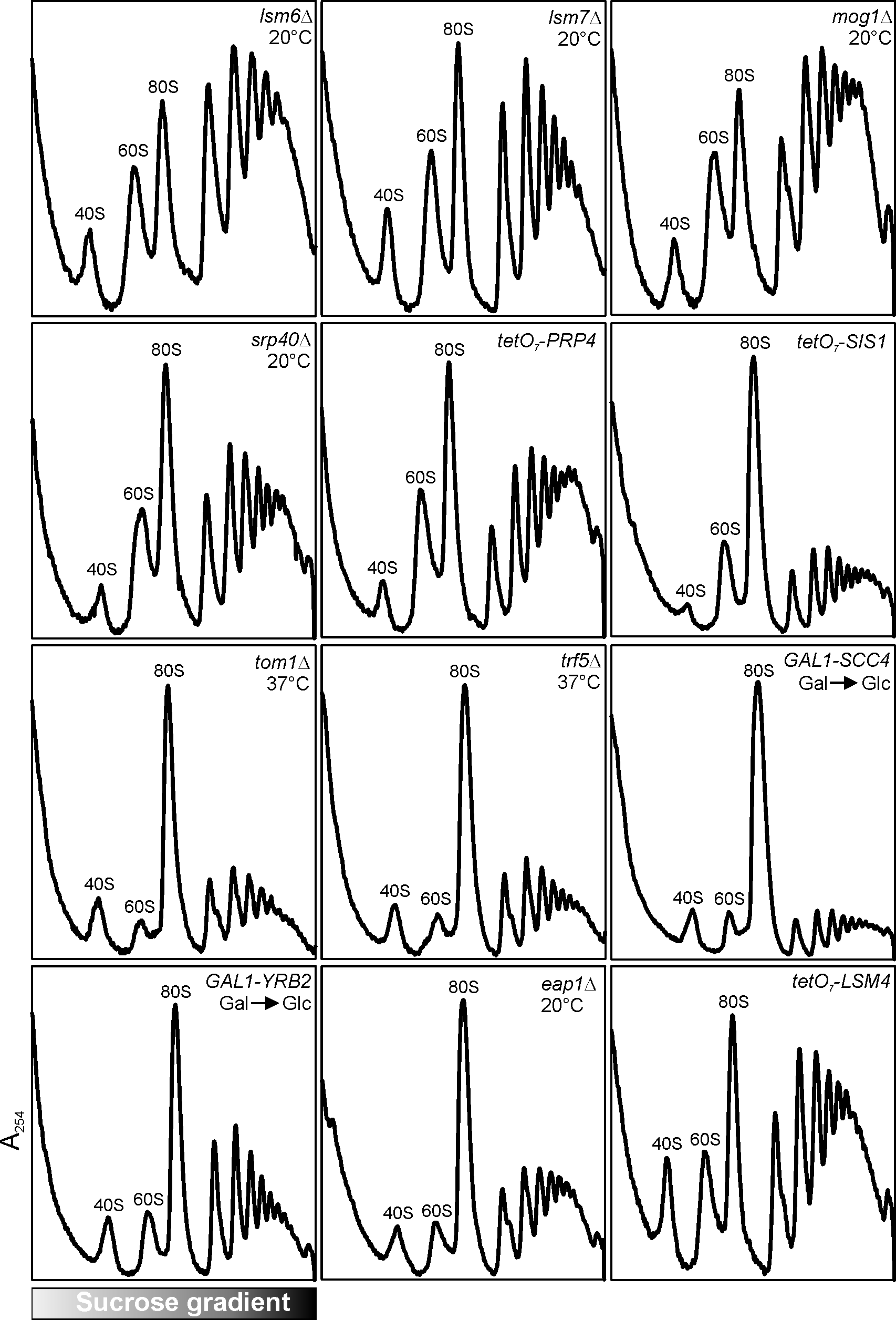

Supplement: Figure S2 — Polysomal profiles of mutants with slightly imbalanced ribosomal subunits. Mutants were cultured at 30°C unless otherwise indicated in the figure. Peaks corresponding to 40S and 60S ribosomal subunits and 80S mono-ribosomes in the polysome profiles are labeled. (0.20 MB TIF) [file pbio.1000213.s002.tif]

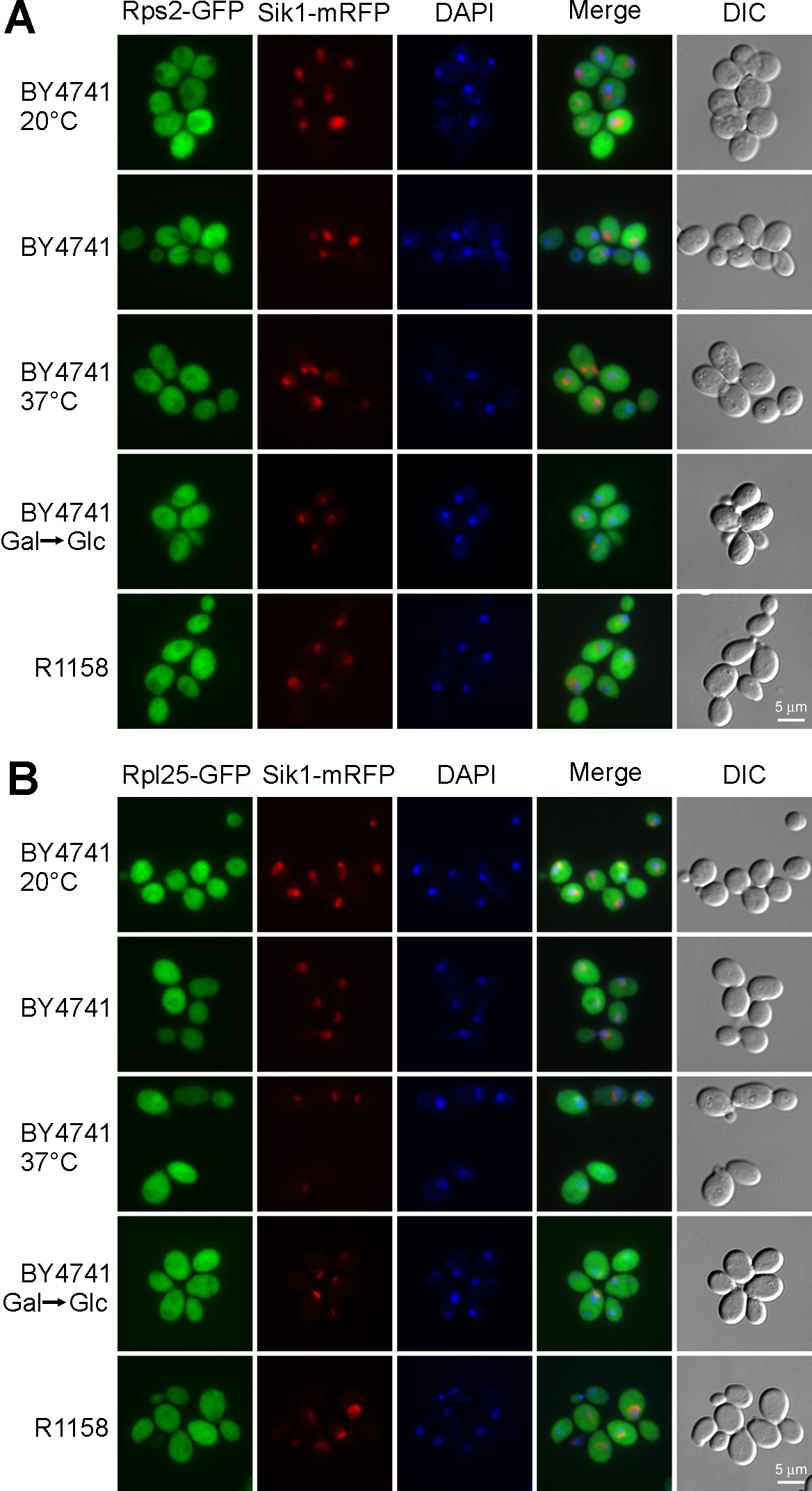

Supplement: Figure S3 — Ribosomal subunit nuclear export assay in wild-type yeast strains under different conditions. (A) Ribosomal small subunits mainly localize to the cytoplasm of wild-type strains under assayed conditions. Rps2-GFP and Sik1-mRFP were used as the reporters for 40S small subunits and the nucleolus, respectively. DAPI was used to stain the nucleus. BY4741 is the control strain for the deletion mutants and the strains with GAL1-promoter controlled alleles. R1158 is the control strain for the strains with tetO7-promoter controlled alleles. The strains were cultured at 30°C unless otherwise indicated in the figure. The white scale bar at the bottom-right corner represents 5 µm. (B) Ribosomal large subunits mainly localize to the cytoplasm of wild-type strains under assayed conditions. Rpl25-GFP was used as the reporter for 60S large subunits. (1.63 MB TIF) [file pbio.1000213.s003.tif]

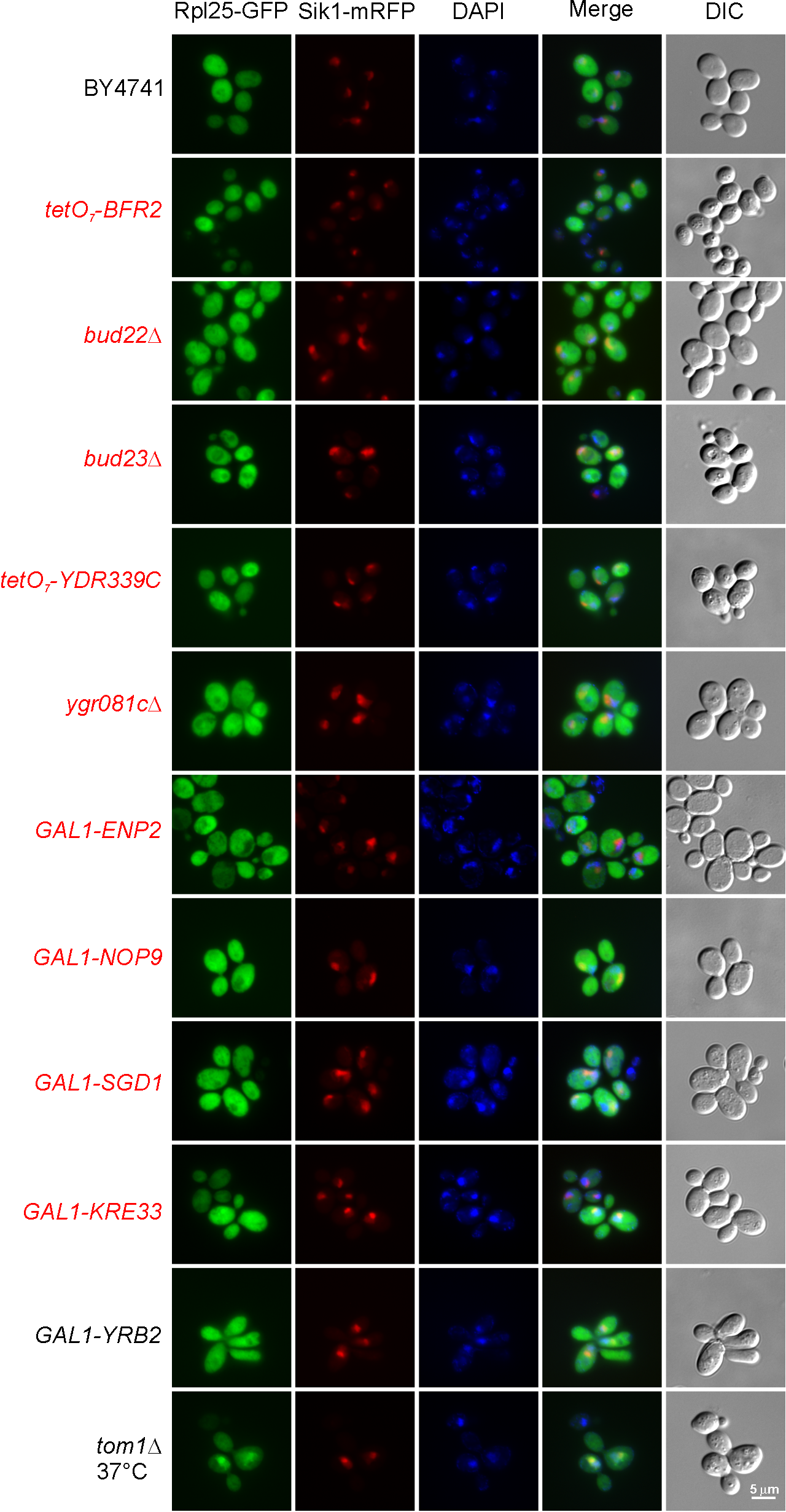

Supplement: Figure S4 — Ribosomal 60S subunit nuclear export was largely unaffected in mutants with 40S nuclear export defects ( Figure 7A ). BY4741 is the representative control strain. Strains with GAL1-promoter controlled alleles or tetO7-promoter controlled alleles were cultured as described in Text S1. Labels in this figure conform to Figure S3. (2.04 MB TIF) [file pbio.1000213.s004.tif]

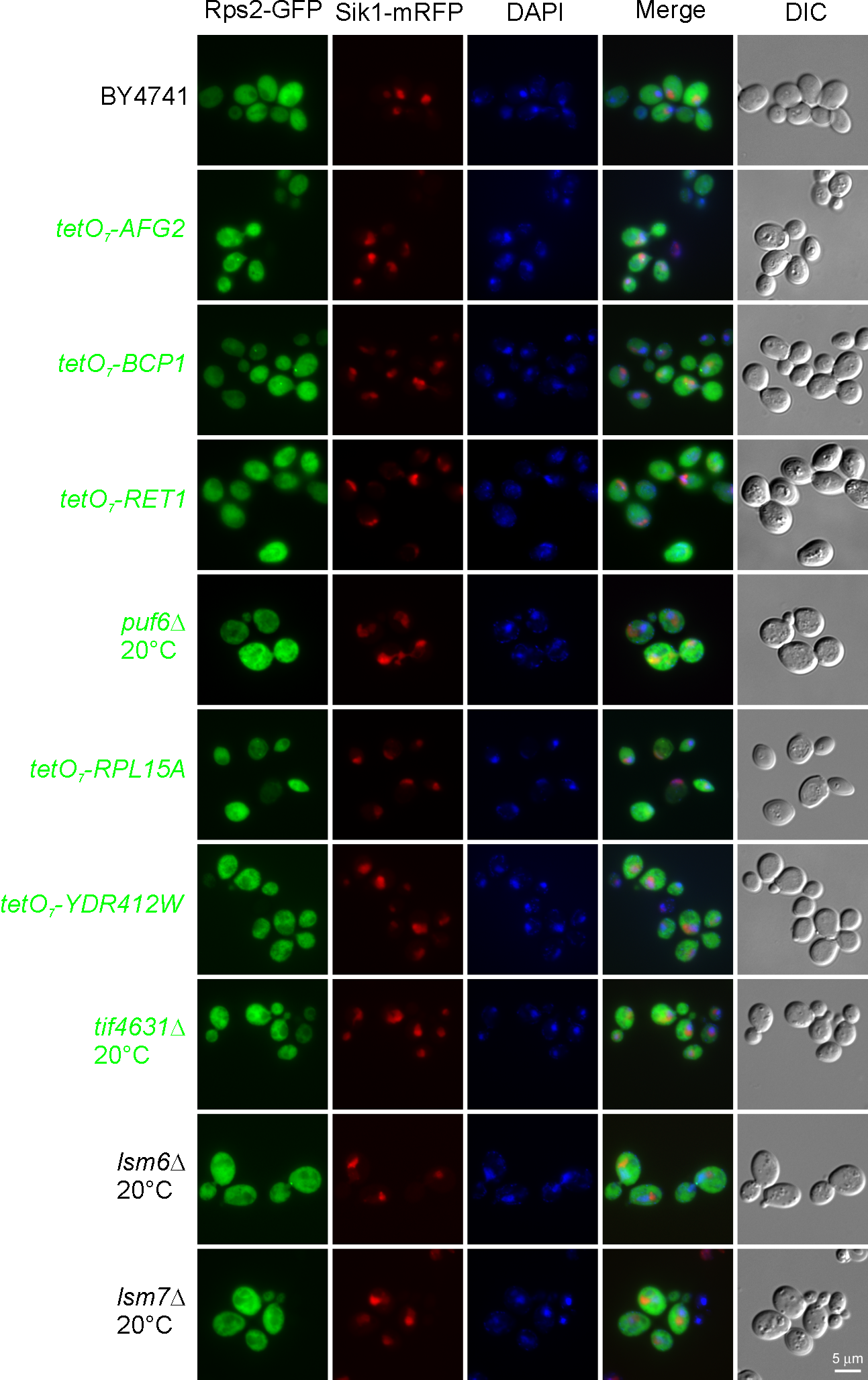

Supplement: Figure S5 — Ribosomal 40S subunit nuclear export was largely unaffected in mutants with 60S nuclear export defects ( Figure 7B ). BY4741 is the representative control strain. Strains with GAL1-promoter controlled alleles or tetO7-promoter controlled alleles were cultured as described in Text S1. Labels in this figure conform to Figure S3. (1.65 MB TIF) [file pbio.1000213.s005.tif]

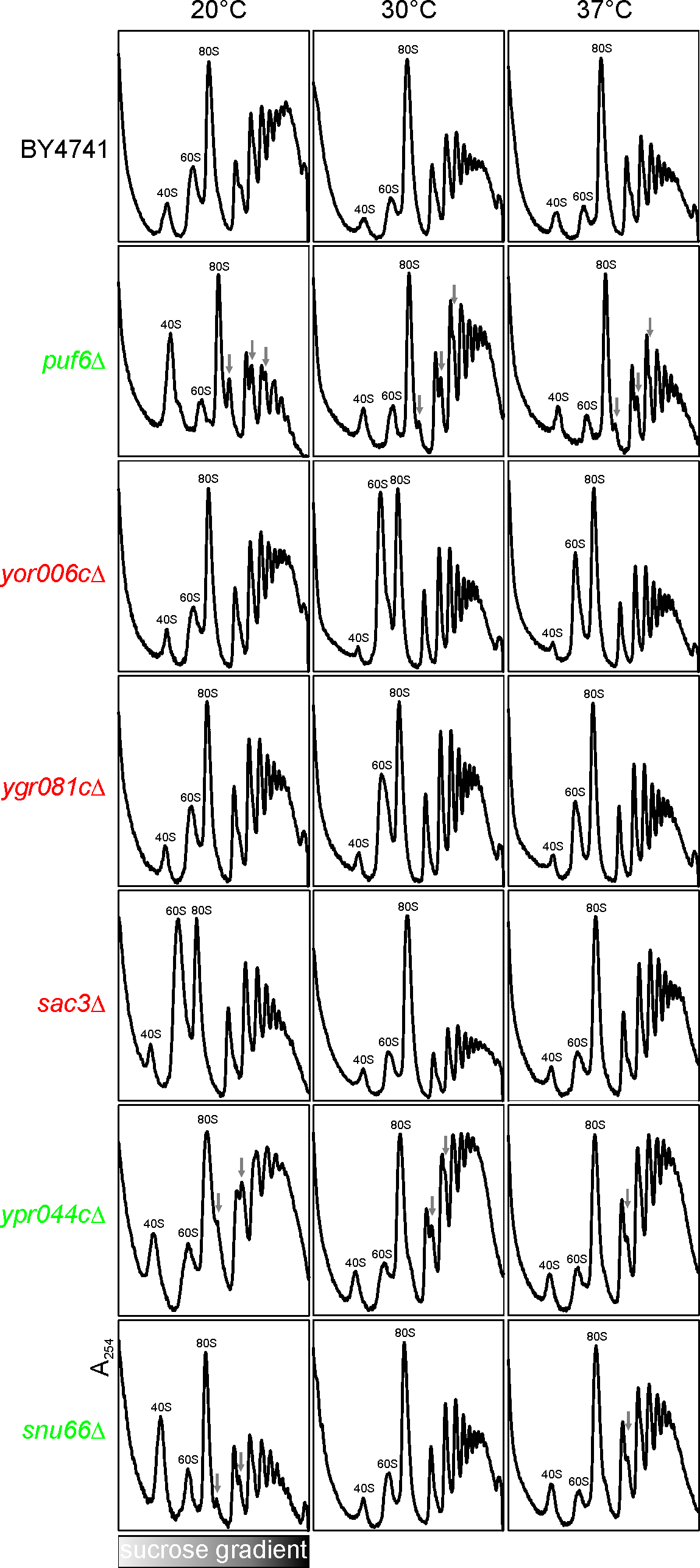

Supplement: Figure S6 — Polysomal profiles of mutants cultured at different temperatures. Strains were cultured at 20°C, 30°C, and 37°C. Peaks corresponding to 40S and 60S ribosomal subunits and 80S mono-ribosomes in the polysome profiles were labeled. Gray arrows indicate the halfmer polysomes. Different mutants showed different temperature-dependent defects in the synthesis of ribosomal subunits. (0.35 MB TIF) [file pbio.1000213.s006.tif]
