# Supplementary material for: Rational Extension of the Ribosome Biogenesis Pathway Using Network-Guided Genetics
Source: PLoS Biol. 2009 Oct 6;7(10):e1000213. doi: 10.1371/journal.pbio.1000213 (PMC2749941; doi:10.1371/journal.pbio.1000213)
Supplement: Text S1 — Supplemental methods and references. (0.06 MB DOC) [file pbio.1000213.s010.doc]

### Supplemental Methods

#### Plasmid Construction and Transformation into Yeast

The BamHI-digested fragments from pFL45/ACT and pFL45/ACT/yU24 [1] were ligated with BamHI-digested pRS413 [2] to result in pRS413-ACT and pRS413-ACT/U24, respectively. pRS413-ACT carries the yeast actin gene with a modified intron [1] under the control of the promoter and terminator of the yeast alcohol dehydrogenase gene. pRS413-ACT/U24 contains the U24 intron sequence within the intron region of the actin gene.

The full-length *ASC1* gene with its own promoter (upstream 624 nucleotides of *ASC1*) and terminator (downstream 149 nucleotides of *ASC1*) sequences were amplified from yeast genomic DNA by using primers ASC-F1 (5’-GCAGCCCGGGGGATCCTTGGAC AATAACCTCAGCGA-3’) and ASC1-R1 (5’-TAGAACTAGTGGATCCTAATGATA ACCTTTCTTTCATTACTTCG-3’). The full-length *ASC1* gene was cloned into pRS416 at the BamHI site using In-Fusion PCR cloning (Clontech). The resulting plasmid pRS416-ASC1 was verified by sequencing.

The upstream 624 nucleotides and the first exon sequence of *ASC1* were amplified by PCR from yeast genomic DNA by using primers ASC-F1 and ASC1-MR (5’-CAATTTGGAATTGGTTTAAGTTCCAAGCCTTAACCATTTTGTCGTTACCGG-3’). The second exon sequence of *ASC1* was amplified by PCR from yeast genomic DNA by using primers ASC1-MF (5’-CCGGTAACGACAAAATGGTTAAGGCTTGGAACT TAAACCAATTCCAAATTG-3’) and ASC1-R1. The first exon fragment including its upstream 624 nucleotides and the second exon fragment were mixed as template to amplify the full length ORF of *ASC1* and its upstream 624 nucleotides by using primers ASC1-F1 and ASC1-R1. The full length ORF of *ASC1* and its upstream 624 nucleotides was inserted into the BamHI site of pRS416 at by In-Fusion PCR cloning (Clontech). The resulting plasmid pRS416-ASC1ORF was verified by sequencing.

Plasmids were transformed into yeast cells using the lithium acetate/single-strand carrier DNA/polyethylene glycol method [3].

#### Strain Construction

To construct strains with GAL1-promoter controlled essential alleles, PCR fragments amplified from pFA6a-KanMX6-GAL1 [4] by gene-specific oligonucleotides (listed in Table S2) were transformed into BY4741 (*MATa his3Δ1 leu2Δ0 met15Δ0 ura3Δ0*). Clones with the inserted *kan^r^* gene and GAL1 promoter before the start codon of each gene *via* homologous recombination were selected and confirmed by PCR.

*GAL1-PAP2* was constructed in strain BY4742 (*MATα his3Δ1 leu2Δ0 lys2Δ0 ura3Δ0*) by inserting the HIS3 gene and the GAL1 promoter before the start codon of *PAP2* *via* homologous recombination. The MATa *trf5ΔGAL1-PAP2* strain was constructed by mating the strain MATa *trf5Δ::kanMX6* with MATα *His3MX6:GAL1-PAP2* to obtain a heterozygote, followed by tetrad dissection and selection to obtain the double mutant.

#### Sucrose Density Gradient Sedimentation

200 μg/ml cycloheximide (Sigma) was added to each culture to arrest translation. Cultures were immediately cooled with ice and all subsequent steps were performed on ice or at 4°C. Cells were collected by centrifugation at 3000g for 5 minutes and washed once with lysis buffer (20 mM Tris pH 7.4, 20 mM KCl, 5 mM MgCl_2_, 200 μg/ml cycloheximide, 12 mM β-mercaptoethanol). The cell pellet was resuspended in the lysis buffer with protease inhibitors (2 μg/ml leupeptin, 2 μg/ml aprotinin, 1 μg/ml bestatin, 1 μg/ml pepstatin A, 1mM PMSF) and lysed by vortexing with glass beads. Crude lysates were clarified by centrifuging at 15,000g for 10 minutes. Fifteen OD_260_ units of the supernatant were loaded onto a continuous 7% to 47% sucrose gradient. After centrifuging for 2.5-hr at 40,000 rpm in a Beckman SW40 rotor, the sucrose gradient was fractionated and absorbance at 254 nm was measured (ISCO fractionator).

#### Mass Spectrometry

Each trypsin digested sample was diluted with 94.9% water, 5% acetonitrile, 0.1% formic acid, and filtered with a Microcon YM-10 filter (Millipore). Each peptide mixture was first separated by a strong cation exchange column with a four-step salt elution: 5, 20, 60, and 900 mM ammonium chloride. Each salt eluate was loaded directly onto a reverse phase C18 column. After washing, peptides were eluted by a 125-min continuous gradient from 5%-50% acetonitrile, and analyzed online with an electrospray ionization ion trap mass spectrometer (ThermoFinnigan DecaXPplus). Each sample was analyzed three times at different parent ion mass/charge (m/z) ranges (300-650, 650-900, and 900-1500). For each parent ion mass spectrum, the top five spectra were selected for fragmentation by collision induced dissociation with helium gas to produce MS/MS spectra. The MS/MS spectra for each fraction were combined and used to search against a database of *Saccharomyces cerevisiae* protein sequences by using the SEQUEST algorithm (Bioworks 3.2, Thermo). Proteins were identified at a 5% false detection rate by using PeptideProphet and ProteinProphet [5].

#### Microscopic Image Analysis

Quantitative image analysis was performed using custom image processing software. The image processing software was written for the MATLAB programming environment and utilized the MATLAB image processing toolbox. The analysis objective was to quantify export defects by determining the relative concentration of GFP-tagged ribosomal subunits in the nucleus and nucleolus relative to the concentration in the cytoplasm. This required detection of yeast cell boundaries as well as detection of the nucleus and nucleolus. The general approach was as follows: we used a composite image of all fluorescent channels to locate cells, then separated clumps of cells into individual cells using a seeded watershed segmentation algorithm [6,7]. We identified each cell’s nucleus and nucleolus using automated cell-by-cell thresholding on the DAPI and TRITC fluorescent images, respectively. The median intensity per pixel for GFP in each subcellular region was then calculated and used to estimate the relative abundance of GFP-tagged ribosomal subunits.

Prior to image analysis, a small diameter median filter was employed in order to remove speckles and shot noise. A background correction was applied to each fluorescent image to compensate for uneven illumination. We estimated the background by applying a large diameter morphological opening filter to each image, which removed all cells in the field of view but preserved underlying variations in the background.

Cellular regions were identified using Kittler and Illingworth’s minimum error thresholding method [8] on a composite fluorescent image that combined the three available fluorescent channels (DAPI, FITC and TRITC). This operation resulted in a binary image that distinguished cellular from non-cellular regions. Cell boundaries were then smoothed using a morphological filter, and small objects that were not commensurate with a yeast cell were removed. Additionally, cells that intersected image boundaries were removed.

Cell clumps were segmented into individual cells using a seeded watershed technique [6,7] in which the nucleolar region defined by Sik1-mRFP was used as a seed. Infrequently the nucleolar marker was not observed in a cell which resulted in an under-segmentation error. This problem was mitigated by employing an upper cell size limit that usually removed under-segmented cell clumps. After cell segmentation, cell boundaries were further refined using a morphological erosion filter. This filter compensated for blurred cell edges that were caused by the presence of out of focal plane light, a common defect of standard fluorescent microscopy. Cells that exhibited extremely low total fluorescent intensity in the GFP channel were removed.

After individual cells were identified, the subcellular regions were identified using automated cell-by-cell thresholding on the DAPI and TRIC fluorescent images. Automated cell-by-cell thresholding is superior to whole field thresholding because it allows for clear identification of regions despite total intensity differences between individual cells. The location of the nucleolus was determined by cell-by-cell thresholding of the TRITC fluorescent image. The location of the nucleus was determined by cell-by-cell thresholding of the fluorescent DAPI image, and assuming the subcellular region with the highest total intensity in each cell was the nucleus. The cytoplasm was then assumed to be all pixels attributed to each cell that were not attributed to the nucleus and nucleolus.

After all cells and subcellular regions were identified, the relative concentration of ribosomal subunits in each region was found by calculating the median intensity per pixel in each of the identified regions for each cell. Then the ratio of this statistic in the nuclear and nucleolar regions relative to the cytoplasm for each cell was calculated. For each strain, the median of ratios of all tested cells was used as an index to evaluate the accumulation of ribosomal subunits in the nucleus or nucleolus.

### Supplemental References

1. Kiss-Laszlo Z, Henry Y, Kiss T (1998) Sequence and structural elements of methylation guide snoRNAs essential for site-specific ribose methylation of pre-rRNA. EMBO J 17: 797-807.

2. Sikorski RS, Hieter P (1989) A system of shuttle vectors and yeast host strains designed for efficient manipulation of DNA in Saccharomyces cerevisiae. Genetics 122: 19-27.

3. Gietz RD, Woods RA (2002) Transformation of yeast by lithium acetate/single-stranded carrier DNA/polyethylene glycol method. Methods Enzymol 350: 87-96.

4. Longtine MS, McKenzie A, 3rd, Demarini DJ, Shah NG, Wach A, et al. (1998) Additional modules for versatile and economical PCR-based gene deletion and modification in Saccharomyces cerevisiae. Yeast 14: 953-961.

5. Nesvizhskii AI, Keller A, Kolker E, Aebersold R (2003) A statistical model for identifying proteins by tandem mass spectrometry. Anal Chem 75: 4646-4658.

6. Gonzalez RC, Woods, R. E., and Eddins, S. L. (2004) Digital Image Processing Using MATLAB; Gonzalez RC, Woods, R. E., and Eddins, S. L., editor: Pearson Prentice Hall, NJ, USA.

7. Vincent L, Soille P (1991) Watersheds in Digital Spaces: An Efficient Algorithm Based on Immersion Simulations. IEEE Transactions on Pattern Analysis and Machine Intelligence 13: 583-598.

8. Kittler J, Illingworth J (1986) Minimum error thresholding. Pattern Recogn 19: 41-47.
